# Vitamin K2 supplementation improves impaired glycemic homeostasis and insulin sensitivity for type 2 diabetes through gut microbiome and fecal metabolites

**DOI:** 10.1186/s12916-023-02880-0

**Published:** 2023-05-05

**Authors:** Yuntao Zhang, Lin Liu, Chunbo Wei, Xuanyang Wang, Ran Li, Xiaoqing Xu, Yingfeng Zhang, Guannan Geng, Keke Dang, Zhu Ming, Xinmiao Tao, Huan Xu, Xuemin Yan, Jia Zhang, Jinxia Hu, Ying Li

**Affiliations:** 1 Department of Nutrition and Food Hygiene, the National Key Discipline, School of Public Health, Harbin Medical University, Harbin, China; 2 Department of Nutrition, the Second Affiliated Hospital of Harbin Medical University, Harbin, China; 3 Department of Endocrinology, the First Affiliated Hospital of Harbin Medical University, Harbin, China

**Keywords:** Vitamin K2, Type 2 diabetes, Gut microbiota, Fecal metabolites, Glycemic homeostasis

## Abstract

**Background:**

There is insufficient evidence for the ability of vitamin K2 to improve type 2 diabetes mellitus symptoms by regulating gut microbial composition. Herein, we aimed to demonstrate the key role of the gut microbiota in the improvement of impaired glycemic homeostasis and insulin sensitivity by vitamin K2 intervention.

**Methods:**

We first performed a 6-month RCT on 60 T2DM participants with or without MK-7 (a natural form of vitamin K2) intervention. In addition, we conducted a transplantation of the MK-7-regulated microbiota in diet-induced obesity mice for 4 weeks. 16S rRNA sequencing, fecal metabolomics, and transcriptomics in both study phases were used to clarify the potential mechanism.

**Results:**

After MK-7 intervention, we observed notable 13.4%, 28.3%, and 7.4% reductions in fasting serum glucose (*P* = 0.048), insulin (*P* = 0.005), and HbA1c levels (*P* = 0.019) in type 2 diabetes participants and significant glucose tolerance improvement in diet-induced obesity mice (*P* = 0.005). Moreover, increased concentrations of secondary bile acids (lithocholic and taurodeoxycholic acid) and short-chain fatty acids (acetic acid, butyric acid, and valeric acid) were found in human and mouse feces accompanied by an increased abundance of the genera that are responsible for the biosynthesis of these metabolites. Finally, we found that 4 weeks of fecal microbiota transplantation significantly improved glucose tolerance in diet-induced obesity mice by activating colon bile acid receptors, improving host immune-inflammatory responses, and increasing circulating GLP-1 concentrations.

**Conclusions:**

Our gut-derived findings provide evidence for a regulatory role of vitamin K2 on glycemic homeostasis, which may further facilitate the clinical implementation of vitamin K2 intervention for diabetes management.

**Trial registration:**

The study was registered at https://www.chictr.org.cn (ChiCTR1800019663).

**Supplementary Information:**

The online version contains supplementary material available at 10.1186/s12916-023-02880-0.

## Background

Diabetes mellitus remains a major health threat worldwide because of its complicated pathogenesis [1]. In recent years, it has become evident that type 2 diabetes mellitus (T2DM) is strongly associated with gut microbiota dysbiosis [2] through multiple mechanisms, including alterations in metabolites produced via gut microbiota saccharolysis or proteolysis, increased gut permeability and perturbation of bile acid metabolism [3]. Altered composition of the gut microbiota has been observed in T2DM patients and prediabetes [4, 5], whereas fecal microbiota transplantation (FMT) from healthy donors to metabolic syndrome patients improved glycemic control and insulin sensitivity [6].

It should be noted that the existing microbiota and the host have established a high level of fitness through long-standing interactions, which is a non-negligible obstacle to the long-term efficacy of existing FMT and probiotic supplementation methods [7]. As the available supplementation options almost involve oral ingestion, the supplemented microbes must remain active as they pass through the digestive tract prior to colonization, which is difficult [8]. In contrast, substances that can maintain the homeostasis of the gut microbiota, such as prebiotics and postbiotics, may be a more effective and cost-effective choice for diabetes management [9].

In recent years, cohort studies and randomized controlled trials (RCTs) have demonstrated the potential benefits of vitamin K2 in insulin sensitivity and glucose metabolism: (1) Beulens et al. investigated the risk of T2DM with vitamin K2 dietary intake according to the FFQ survey in a large prospective study involving 38,094 Dutch adults. It had been observed that vitamin K2 intake was linearly and negatively associated with the risk of T2DM (*P* = 0.038), with a HR of 0.93 (0.87–1.00) per 10 μg increase [10]; (2) Choi et al. conducted a placebo-controlled trial in which 33 young males were given 30 mg/d of MK-4 (a form of vitamin K2) or a placebo for a period of 4 weeks. It had been observed that vitamin K2 supplementation significantly increased the insulin sensitivity index (*P* = 0.01) and the disposition index (*P* < 0.01), but these changes did not occur in the placebo group [11]. Animal and cellular studies have also shown that vitamin K2 can improve glycemic homeostasis by regulating the circulating levels of osteocalcin (a vitamin K-dependent calcium-binding protein) [12] and glucagon-like peptide-1 (GLP-1) [13] and improving lipid parameters [14]. Importantly, a recent evidence has demonstrated that the microbiota composition significantly changes when diet-derived vitamin K is insufficient in the gut environment [15], although the microbiota is capable of producing vitamin K2 on its own. Although previous studies have provided valuable insights into the effects of vitamin K2 on glycemic homeostasis and gut microbiota, vitamin K2 is poorly understood as a metabolic intervention that regulates blood glucose by acting on the gut microbiota.

We hypothesized that vitamin K2 may indirectly improve glycemic homeostasis by acting as a gut stabilizer through the microbiota. To address the above questions and confirm the effects of vitamin K2 supplementation on glycemic homeostasis and the gut microbiota, we first conducted a double-blind randomized controlled MK-7 intervention for 6 months in community-recruited T2DM patients, followed by 16S rRNA sequencing and metabolomics analysis to clarify the alterations in clinical characteristics and the compositional and functional shifts in the gut microbiota. In addition, we performed a functional exploration of the effects of vitamin K2 on the microbiota in mice by using the FMT method to validate the role of the altered gut microbiota and fecal metabolites in glucose metabolism and insulin sensitivity.

## Methods

Methods of the clinical parameter measurements, biological sample collection [16], biochemical index testing, 16S rRNA [17–21] and transcriptome sequencing, targeted metabolomics detection [22], RT-qPCR (primer sequences are in Additional file 1: Table S1), pathological examination, and immunohistochemistry are enclosed in Additional file 1.

### Subject recruitment and study design

**Fig. 1:**
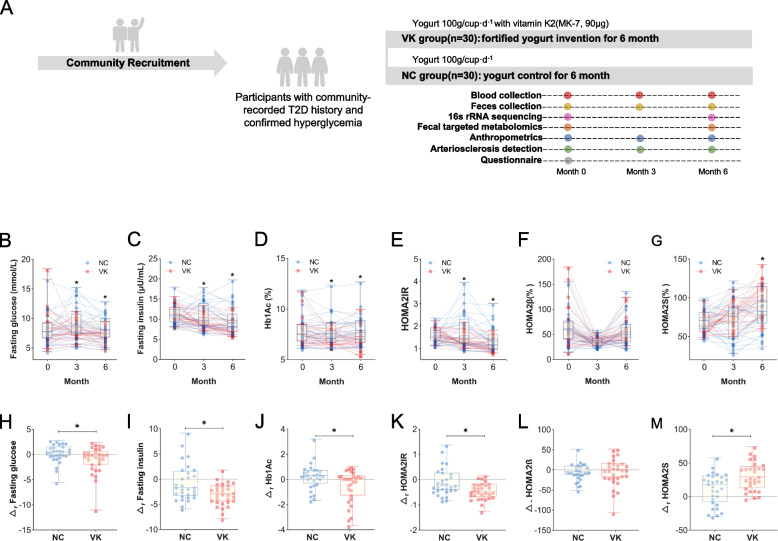
Study design and the improvement of glycemic indicators. **A** Schematic workflow of the study design. **B**–**G** Boxplots showing the dynamic changes of **B** fasting glucose, **C** fasting insulin, **D** Hb1Ac, and **E**–**G** HOMA2 model at 0, 3, and 6 months of MK-7 intervention within all subjects (NC group = 30 and VK group = 30). Lines match the same subject at different time points. *P < 0.05 by ANCOVA controlling for corresponding baseline values. **H**–**M** The relative change of **H** fasting glucose, **I** fasting insulin, **J** Hb1Ac, and **K**–**M** HOMA2 model over 6 months of MK-7 intervention. *P < 0.05 by independent Student’s *t* test

T2DM subjects were recruited from 4 local communities in Harbin (Heilongjiang, China) through flyers and on-site advertisements. Inclusion was limited to subjects with a definite T2DM history and confirmation of diagnosis by physicians. The exclusion criteria were severe cardiovascular events, recurrent infections, any surgery, gastrointestinal disease, organ failure, use of antibiotics or warfarin in the past 3 months, and use of more than 2 kinds of medicines for glycemic control. In addition, participants did not take any vitamin K2 supplements or probiotics. Eligible subjects were randomly assigned to the vitamin K2 supplementation group (referred to as the “VK group” in subsequent analysis, 90 µg MK-7 was added in 100 g yogurt, 1 cup (100 g)/day) or the control group (“NC group,” 100 g yogurt without MK-7 added, 1 cup (100 g)/day). To ensure compliance in the study population and to take into account the characteristics of vitamin K2, we used yogurt instead of a capsule as a good carrier for vitamin K2 [23, 24] and MK-7 as a supplemental form of vitamin K2 with a longer half-life and high bioavailability. There was no difference of the yogurt in appearance or taste between the two groups. Yogurt products were pasteurized to ensure they were free of bacteria and other possible contaminants. Yogurt was allocated by community staff according to labels with the subjects’ names on the yogurt cups, but they had no information about our study design, nor did the subjects. The choice of dose was based on our previous research [25]. The detailed study design, sample calculation, fecal collection method, and implementation process are described in Fig. 1A and Additional file 1.

### Study design for the FMT experiment in mouse model

In the first part of the study, 42 male C57BL/6N mice (Beijing Vital River Laboratory Animal Technology Co., Ltd, China) were randomly divided into 3 groups at 8 weeks of age after 1 week of adaptation: control (“NC group,” *N* = 7), high-fat (“HF group,” *N* = 28, 60% high-fat diet), and HF + MK-7 (“HFVK group,” *N* = 7, 60% high-fat diet). In addition, mice in the HFVK group were administered 50 µg/kg weight MK-7 supplement (low-dose supplementation compared to other animal studies [26, 27]) every other day by oral gavage, and the other two groups were given corresponding amounts of solvent. Considering the single feeding environment of experimental mice and the difference in composition from the human gut microbiota [28], we chose the feces provided by the mice in the same environment as the source of FMT to objectively observe the effects of the gut microbiota after treatment with MK-7. The mice were subjected to an oral glucose tolerance test (OGTT) at week 9 and week 15 of the study.

In the second part of the study, 28 mice in the HF group were further randomly divided into 4 groups in study week 10: the HF group (*N* = 7) was treated as the previous “HF group” in the first part of the study, HF + mixed antibiotics (“HFABX group,” *N* = 7), HF + mixed antibiotics + feces from the NC group (“NCR group,” *N* = 7), and HF + mixed antibiotics + feces from the HFVK group (“VKR group,” *N* = 7). For mixed antibiotic (ABX) treatment, mice were treated with ampicillin (1 g/L, Aladdin, Cat# A105483), metronidazole (1 g/L, Aladdin, Cat# M109874), neomycin (1 g/L, Aladdin, Cat# N109017), vancomycin (500 mg/L, Aladdin, Cat# V301569) and sucralose (0.05 mg/ml, Aladdin, Cat# S107614) in the drinking water for 14 days [29] during weeks 10–12 of the study. From week 13, FMT was conducted once a day for the first 3 days in the first 2 weeks to promote microbiota engraftment and then twice per week thereafter in the last 2 weeks to maintain the effect of the transplant. FMT lasted a total of 4 weeks from week 13 to week 16 [30]. The mice were subjected to an OGTT at week 16 of the study. It should be emphasized that this part had a noninferior experimental design, as germ-free mice and the healthy gut microbiota could protect against diet-induced obesity (DIO) [31].

### Fecal microbiota transplantation

Fresh feces from the NC group and HFVK group were collected in the morning and evening before the day of transplantation and then immediately frozen at − 80 °C. On the day of transplantation, mixed feces from each group were suspended in PBS buffer (vortex, 5 min) at a ratio of 1 (mg): 100 (mL) before centrifugation at 600 × g for 5 min. The final supernatant was used to gavage each mouse (NCR group and VKR group) with a dose of 10 μL/g body weight, and correspondingly, mice in the HFABX group were given solvent.

### Statistical and bioinformatic analysis

All analyses were conducted by using R software version 4.1.1. Detailed statistical and bioinformatic methods are enclosed in the Additional file 1.

Downstream 16S rRNA analyses were conducted by the R package “microeco” and “Tax4fun.” Genus biomarkers between groups were tested by the rank-sum test and Lefse analysis (raw *P* < 0.05 or LDA score > 3). Differences in fecal metabolites were evaluated by using the Wilcoxon rank-sum test and variable importance for the projection score (VIP) (raw *P* < 0.05 or VIP score > 1). Differentially expressed genes (DEGs) were selected under the criteria of │log_2_fold change│ > 1 and *P* < 0.05 to identify intergroup gene expression differences and whole transcriptomes were subjected to gene set enrichment analysis (GSEA) to identify intergroup functional differences by using the R packages “DESeq2” and “clusterProfiler.” All data are presented in figures as mean ± SEM.

All statistical analyses were conducted using two-tailed hypothesis testing.

## Results

### The 6-month MK-7 intervention improved clinical characteristics of glycemia

Eighty eligible subjects were ultimately enrolled in our evaluation and randomized to either the 6-month supervised NC group or VK group, of whom 60 completed the entire study (*n* = 30 in each group) (Fig. 1A; Additional file 1: Fig.S1). It was shown that there was no significant difference between the two groups at baseline (Table 1).

**Table 1:** Clinical characteristics of diabetic participants to 6-month vitamin K2 (MK-7) intervention

Characteristics	0 month			3 months			6 months			*P* value
	NC group	VK group	*P* value^a^	NC group	VK group	*P* value^b^	NC group	VK group	*P* value^b^	Relative change of parameters between groups^c^
Age (years)	62.97 ± 1.67	63.33 ± 1.33	0.864	-	-	-	-	-	-	-
Sex (male, *n*)	16	13	0.438	-	-	-	-	-	-	-
Current smoking (yes, *n*)	3	2	0.533	-	-	-	-	-	-	-
Current drinking (yes, *n*)	9	7	0.448	-	-	-	-	-	-	-
Exercise habit (> 3 times/week, *n*)	22	19	0.432	-	-	-	-	-	-	-
Course of diabetes			0.860	-	-	-	-	-	-	-
5 years	8	9		-	-	-	-	-	-	-
5–10 years	3	2		-	-	-	-	-	-	-
10 years	19	17		-	-	-	-	-	-	-
BMI (kg/m^2^)	25.41 ± 0.59	24.98 ± 0.54	0.598	24.92 ± 0.64	24.76 ± 0.55	0.538	25.36 ± 0.56	25.01 ± 0.66	0.727	0.700
Fat mass (%)	28.63 ± 1.22	28.80 ± 0.85	0.909	28.25 ± 1.24	27.22 ± 1	0.217	29.28 ± 1.22	29.46 ± 0.84	0.960	0.978
Waist (cm)	91.14 ± 1.73	86.77 ± 1.73	0.079	90.66 ± 1.37	88.97 ± 1.5	0.220	91.37 ± 1.7	87.51 ± 1.66	0.957	0.648
Hip circumference (cm)	97.49 ± 1.24	96.13 ± 1.04	0.404	98.29 ± 1.14	99.98 ± 1.21	0.025	102.57 ± 1.16	99.63 ± 1.4	0.154	0.221
Systolic blood pressure (mmHg)	131.87 ± 3.04	132.63 ± 2.7	0.851	131.72 ± 2.5	140.8 ± 4.11	0.054	136.1 ± 2.75	138.36 ± 4.57	0.779	0.834
Diastolic blood pressure (mmHg)	75.7 ± 1.5	76.77 ± 1.92	0.663	75.69 ± 1.54	81.5 ± 1.96	0.022	79.2 ± 1.72	78.68 ± 2.25	0.716	0.628
ABI	1.12 ± 0.02	1.17 ± 0.02	0.093	1.11 ± 0.03	1.19 ± 0.02	0.222	1.14 ± 0.03	1.17 ± 0.02	0.652	0.320
baPWV (cm/s)	1555.52 ± 53.4	1648.43 ± 51.33	0.215	1621.79 ± 70.86	1675.22 ± 48.62	0.662	1755.95 ± 75.21	1768.27 ± 77.08	0.177	0.185
dp-ucMGP (pM)	501.20 ± 33.86	498.43 ± 33.98	0.954	455.32 ± 34.42	365.9 ± 26.37	0.002	454.62 ± 35.06	333.34 ± 26.90	< 0.001	< 0.001
Fasting glucose (mM)	7.89 ± 0.45	8.06 ± 0.49	0.803	8.93 ± 0.51	7.76 ± 0.25	0.018	8.01 ± 0.38	6.98 ± 0.2	0.006	0.048
Fasting insulin (μU/mL)	10.65 ± 0.43	11.13 ± 0.38	0.397	10.59 ± 0.62	9.1 ± 0.31	0.016	9.73 ± 0.61	7.98 ± 0.35	0.006	0.005
HbA1c (%)	7.70 ± 0.26	7.68 ± 0.22	0.955	7.70 ± 0.25	7.10 ± 0.13	0.003	7.84 ± 0.25	7.11 ± 0.19	0.006	0.019
Triglyceride (mM)	2.06 ± 0.34	1.68 ± 0.18	0.318	2.38 ± 0.28	1.71 ± 0.14	0.072	2.23 ± 0.3	1.54 ± 0.11	0.045	0.253
Total cholesterol (mM)	4.76 ± 0.2	4.81 ± 0.17	0.846	5.01 ± 0.17	5.06 ± 0.21	0.964	5.26 ± 0.21	4.75 ± 0.12	0.006	0.012
HDL-c (mM)	1.34 ± 0.05	1.42 ± 0.05	0.254	1.23 ± 0.05	1.39 ± 0.07	0.149	1.27 ± 0.05	1.43 ± 0.06	0.088	0.207
LDL-c (mM)	3.29 ± 0.19	3.33 ± 0.18	0.874	3.71 ± 0.14	3.47 ± 0.18	0.115	3.3 ± 0.17	3.01 ± 0.12	0.071	0.136
AST/ALT	1.32 ± 0.14	1.19 ± 0.14	0.513	1.19 ± 0.11	1.07 ± 0.11	0.634	1.02 ± 0.09	1.05 ± 0.13	0.100	0.084
Uric acid (mM)	357.34 ± 17	328.26 ± 16.64	0.226	350.55 ± 20.75	357.22 ± 21.32	0.057	335.95 ± 19.51	313.41 ± 15.77	0.948	0.696
Creatinine (mM)	66.47 ± 5.99	59.57 ± 2.57	0.294	68.03 ± 5.53	60.8 ± 2.85	0.634	79.17 ± 5.6	71.4 ± 2.68	0.494	0.743
HOMA-IR	1.53 ± 0.06	1.61 ± 0.05	0.311	1.74 ± 0.15	1.36 ± 0.06	0.010	1.4 ± 0.09	1.12 ± 0.05	0.005	0.003
HOMA-IS (%)	68.48 ± 2.65	64.19 ± 2.16	0.214	68.61 ± 4.87	76.88 ± 2.88	0.084	78.65 ± 4.00	93.88 ± 4.00	0.003	0.001
HOMA-β (%)	60.62 ± 5.94	62.36 ± 7.08	0.852	32.51 ± 1.91	35 ± 1.24	0.283	52.76 ± 5.17	54.61 ± 4.01	0.835	0.988
Total energy (Kcal/day)	1392.61 ± 95.43	1226.33 ± 106.23	0.249	-	-	-	-	-	-	-
Carbonhydrate (g)	176.22 ± 13.89	157.96 ± 15.37	0.382	-	-	-	-	-	-	-
Carbonhydrate (% of energy)	51.80 ± 2.57	51.48 ± 1.98	0.922	-	-	-	-	-	-	-
Fat (g)	49.28 ± 4.70	43.18 ± 4.57	0.356	-	-	-	-	-	-	-
Fat (% of energy)	31.01 ± 2.14	31.37 ± 1.67	0.893	-	-	-	-	-	-	-
Protein (g)	61.05 ± 4.97	51.46 ± 4.42	0.154	-	-	-	-	-	-	-
Protein (% of energy)	17.19 ± 0.65	17.15 ± 0.57	0.957	-	-	-	-	-	-	-
Fiber (g)	6.85 ± 0.79	6.87 ± 0.96	0.989	-	-	-	-	-	-	-
Long-term eating habits (in 1 year)			0.466	-	-	-	-	-	-	-
Bland	10	15		-	-	-	-	-	-	-
Normal	17	11		-	-	-	-	-	-	-
Fatty and salty	2	1		-	-	-	-	-	-	-
Vegan	1	1		-	-	-	-	-	-	-
Eating habit of natto (yes, *n*)	1	3	0.280	-	-	-	-	-	-	-
Eating habit of cheese (yes, *n*)	1	4	0.156	-	-	-	-	-	-	-
Hypoglycemic agents										
Metformin (yes, *n*)	30	30	-	-	-	-	-	-	-	-
Insulin (yes, *n*)	4	5	0.734	-	-	-	-	-	-	-
Sulfonylureas (yes, *n*)	5	3	0.456	-	-	-	-	-	-	-

NC group (*n* = 30) and VK group (*n* = 30). Data are shown as mean ± SEM or frequency

*BMI* Body mass index, *ABI* Ankle bronchiole index, *baPWV* Brachial-ankle pulse wave conduction velocity, *HDL-c* High-density lipoprotein cholesterol, *LDL-c* Low-density lipoprotein cholesterol

^a^Calculated by independent Student’s *t* test or chi-square test or Fisher’s exact test

^b^Calculated by ANCOVA controlling for corresponding baseline values

^c^Calculated the difference of relative change (= follow-up − baseline) during the whole intervention by independent Student’s *t* test

After the 6-month intervention, a significant decrease in serum dp-ucMGP in the VK group compared to the NC group indicated that the circulating levels of vitamin K2 in the body had been significantly increased (VK_change_ =  − 165.10 ± 25.30 pmol/L, *P* < 0.001, Table 1) [32]. As our primary outcome, notable reductions of 13.4%, 28.3%, and 7.4% in fasting serum glucose (VK_change_ =  − 1.08 ± 0.49 mmol/L, *P* = 0.048), insulin (VK_change_ =  − 3.15 ± 0.39 μU/mL, *P* = 0.005), and Hb1Ac levels (VK_change_ =  − 0.57 ± 0.23%, *P* = 0.019) were observed in the VK group, together with modest improvements in HOMA index and lipid parameters (Fig. 1B–M, Table 1). However, body mass and the arteriosclerosis index showed no significant differences within or between groups (Table 1). Given the importance of the gut microbiota in glycemic homeostasis, we next investigated the possibility of its involvement in the metabolic effects of vitamin K2 intervention.

### Shifted gut microbiota and reinforced coabundance network after 6 months of MK-7 intervention

**Fig. 2:**
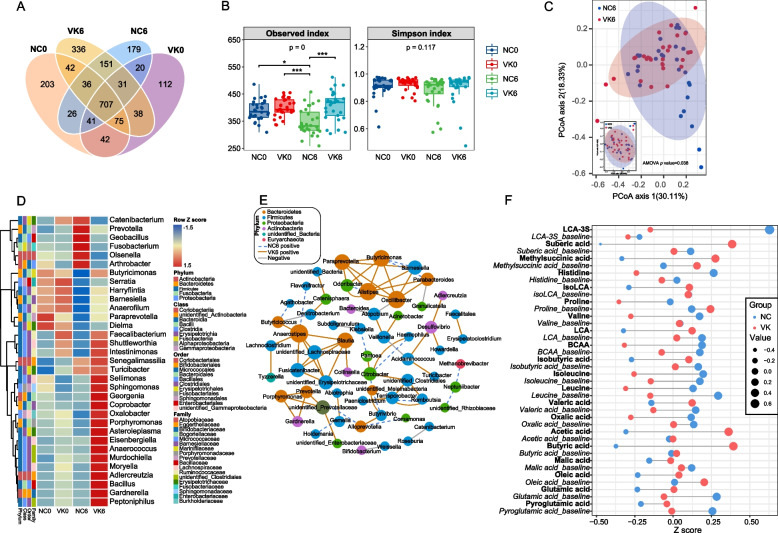
MK-7 intervention promotes differential alterations of gut microbiota and fecal metabolites. **A**–**D** Alterations of gut microbiota over 6-month MK-7 intervention. **A** Overview of the observed amount of OTUs. **B** Alpha diversity measured by the observed index and Simpson index and *P < 0.05, ** P < 0.01, ***P < 0.001 by one-way ANOVA. **C** PCoA based on weighted Unifrac distance showed different taxonomic compositions between NC and VK groups at a terminal time point, and no significance was shown at baseline (lower left corner). *P* value was measured by AMOVA. **D** Heatmap showing the significantly differential microbial genus (*P* < 0.05 or LDA > 3) after 6-month MK-7 intervention accompanied with their corresponding baseline status. The colors changing from blue to red indicate higher relative abundance. **E** Coabundance network at terminal time point. The edges represent significant Pearson correlations of > 0.6 or < -0.6 between genera. Each node represents a genus and is colored based on the affiliated phylum. After 6-month MK-7 intervention accompanied by their corresponding baseline status. Node is based on the degree of connectivity. Blue dashed and orange edges indicate positive correlations in the NC group and VK group and gray edges indicate negative correlations. **F** Dumbbell chart showing the significantly differential fecal metabolites (*P* < 0.05 or VIP > 1) after 6-month MK-7 intervention accompanied with their corresponding baseline status

16S rRNA sequencing of fecal samples revealed that more taxa were observed at termination than at baseline (1682 OTUs vs 1373 OTUs) after the 6-month intervention (Fig. 2A). Although the evenness of the microbiota was not different among groups (Simpson index), the VK6 group (VK group at month 6) showed a protective effect on the richness when compared to the NC6 group (NC group at month 6) (observed index, *P* < 0.001, Fig. 2A, B). In addition, the beta diversity of the microbial community composition, evaluated by the weighted UniFrac distance, showed a significant difference between the NC6 group and the VK6 group after the 6-month MK-7 intervention (all *P* < 0.05, Fig. 2C, Additional file 1: Fig.S3A).

At the phylum level, compared to the baseline status, the ratio of *Firmicutes* and *Bacteroidetes* (F/B) was significantly increased in the NC6 group (NC0 = 4.95 ± 0.96, NC6 = 11.22 ± 2.18, *P* = 0.012), but there was no difference between the VK6 and VK0 groups (VK0 = 4.65 ± 1.10, VK6 = 7.41 ± 1.76,* P* = 0.194) (Additional file 1: Fig.S3B). Although we did not observe the difference between the VK group and NC groups at month 6 (*P* = 0.187), there was a significant positive correlation between the change in serum dp-ucMGP and the change in F/B value in all participants (r_Pearson_ = 0.33, *P* < 0.01, Additional file 1: Fig.S3B), indicating that the improvement nutritional status of circulating MK-7 is related to lower F/B value. Furthermore, the microbiota profile showed that the relative abundance of the genera *Faecalibacterium*, *Intestinimonas*, and *Anaerofilum* (order *Clostridiales*); genera *Butyricimonas*, *Barnesiella*, and *Paraprevotella* (order *Bacteroidales*); and the genera *Dielma* and *Turicibacter* (order *Erysipelotrichales*) was significantly increased in the VK6 group compared to the NC6 group (Fig. 2D). Although the genera *Barnesiella*, *Paraprevotella*, and *Dielma*, showed a decreasing trend throughout the study in both the NC and VK groups, the MK-7 intervention significantly delayed the decline of these genera (Fig. 2D). From an overall view of the family level, *Ruminococcaceae*, *Lachnospiraceae*, and *Bacteroidaceae* were relatively dominant families in the VK6 group compared to NC6, whereas *Enterobacteriaceae* was a dominant family in the NC6 group (Additional file 1: Fig.S3C). Moreover, coabundance network analysis suggested increased density and enhanced interactions in the microbial community after MK-7 intervention compared to baseline or the corresponding NC group (Fig. 2E; Additional file 1: Fig.S3D).

### Distinct functional enrichment and microbial metabolites between the MK-7 intervention and negative control

When comparing the fecal metabolites at baseline and at the end of the study, we observed significant differences in dynamic alterations between the two groups (OPLSDA, Additional file 1: Fig.S3E). Notably, a significant decrease in total branched-chain amino acids (BCAAs) and histidine concentrations was observed after 6 months of intervention, as well as an increase in lithocholic acid (LCA) and short-chain fatty acids (SCFAs) in fecal samples (Fig. 2F). Other changes were mainly related to energy metabolism, such as organic acids or long-chain fatty acids.

Similar to the differences in fecal metabolites, functional enrichment of the microbiota showed a relative enhancement of the capacity for BCAA biosynthesis and amino acid-related enzymes in the NC6 group, although the enhancement of the biosynthesis of aromatic amino acids was only observed in enrichment analysis. On the other hand, MK-7 intervention seemed to involve gut microbiota protein glycosylation reactions, secondary metabolite biosynthesis, and carbohydrate and amino acid metabolism, such as glycosylphosphatidylinositol anchor biosynthesis, flavone and flavonol biosynthesis, glycan biosynthesis and metabolism, and amino sugar and nucleotide sugar metabolism (Additional file 1: Fig.S3F). These results suggested there was a potential association between MK-7 and microbiota and co-metabolites.

### Associations of gut microbiota and fecal metabolites with changes in clinical parameters induced by MK-7 intervention

**Fig. 3:**
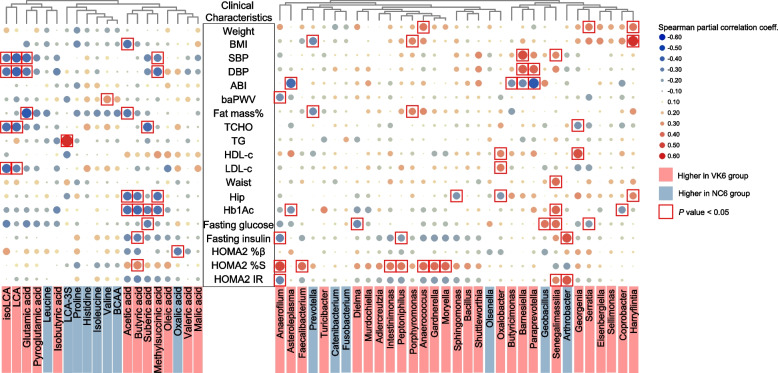
Alterations of microbial genera and fecal metabolites are associated with improvements of clinical characteristics. Heatmap of the Spearman’s rank correlation coefficients between different clinical characteristics and microbial genera and fecal metabolites at terminal time point caused by MK-7 intervention after adjustment for corresponding clinical baseline status

After adjustment for the corresponding status in baseline, we found several strong associations between alterations in genera and fecal metabolites with improvements in glycemic parameters and a cluster of metabolic parameters in the VK group after a 6-month MK-7 intervention. SCFAs and genera whose relative abundance was higher in the VK6 group were associated with reductions in serum Hb1Ac, glucose, insulin and insulin resistance (Fig. 3). Some of these changes, such as *Anaerofilum*, *Asteroleplasma*, *Paraprevotella*, LCA, glutamic acid, and methysuccinic acid, which were found increased in the VK6 group were related to the reduction of lipid parameters such as fat mass, total cholesterol (TCHO), LDL-c, hip circumference as well as blood pressure and arteriosclerosis (Fig. 3). Taken together, changes in these genera as well as fecal metabolites with MK-7 intervention may provide a biological basis for the improvement in clinical indicators in T2DM participants.

### Long-term MK-7 gavage and short-term FMT validate the benefits of the MK-7-regulated microbiota for ameliorating fat accumulation and glucose tolerance in DIO mouse model.

**Fig. 4:**
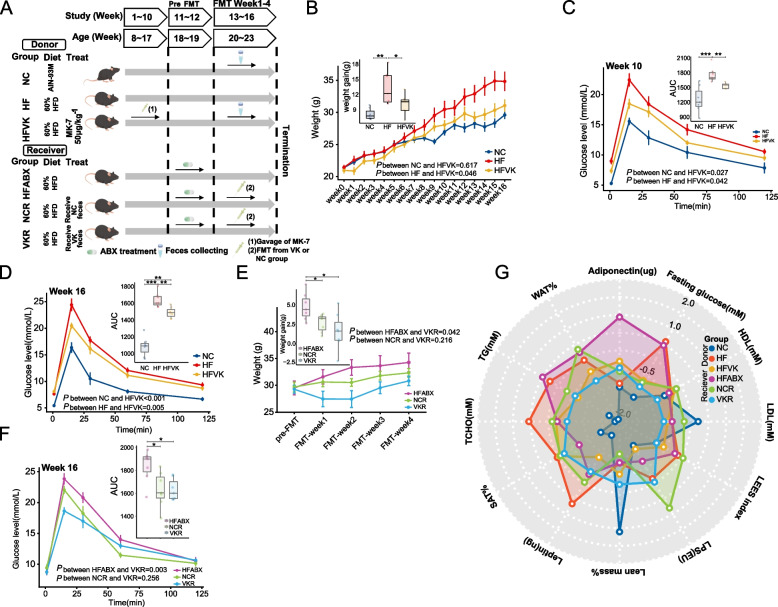
MK-7 intervention and MK-7-regulated microbiota transplantation ameliorate fat accumulation and glucose tolerance. **A** Schematic workflow of the study design for MK-7 intervention and fecal microbiota transplantation. **B**–**D** Overall trends and relative changes in weight gain are shown in **B** and OGTT with the area under the curve (AUC) at week 10 and week 16 are showed in **C**–**D** in donor mice. **E**–**F** Overall trends and relative changes in weight gain after the first FMT are shown in **E** and OGTT with AUC at week 16 are shown in **F** in receiver mice. **G** Radar chart demonstrates the biochemical indicators by using Z-scores in donor and receiver mice. *N* = 7 mice/group. *P < 0.05, ** P < 0.01, ***P < 0.001 by one-way ANOVA with Bonferroni adjustment or two-way repeated-measures ANOVA

To further explore the causal relationship between the MK-7-regulated microbiota and the changes in glucose tolerance and fat accumulation induced by MK-7 intervention, we first performed a 16-week supplementation study in mice fed a 60% high-fat diet and then performed a 4-week FMT study by transplanting feces from donor mice into antibiotic-treated mice (Fig. 4A). As we expected, after both 10 study weeks and 16 study weeks, significantly lower weight gain and better glucose tolerance were observed in NC mice (all *P* < 0.05) and VK mice (all *P* < 0.05) than in HF mice (Fig. 4B–D). Importantly, at 4 weeks after the first FMT, mice colonized with the microbiota from VK mice and NC mice showed reduced weight gain and improved OGTT results compared to HFABX mice (all *P* < 0.05, Fig. 4E–F). Intriguingly, although the glucose tolerance of VK mice (fed a 60% high-fat diet) appeared to be inferior to that of NC mice (fed a standard AIN-93 M diet), the effect of the MK-7-regulated microbiota was comparable to that of the microbiota from lean donors.

Upon further comparison of biochemical markers, the MK-7-regulated microbiota significantly improved fasting glucose, triglyceride (TG), adiponectin, and endotoxin levels and appeared to be able to slightly normalize the percentage of white adipose tissue (WAT%) and lean mass in mice with long-term high-fat feeding (Fig. 4G, Additional file 1: Fig.S4, Additional file 1: Table S2).

### The MK-7-regulated gut microbiota showed shifts in composition and function in mouse model

When we once again detected alterations in the gut microbiota to validate whether MK-7 could independently affect the microbiota (eliminating the potential effects of yogurt and antidiabetics on gut microbiota in the previous RCT study), we found no significant changes in the number of taxa and common species among the three groups, except for the significant difference in overall composition between the NC group and the other two groups (*P* < 0.001) (Additional file 1: Fig.S5A-S5D). Considering the single rearing and feeding environment of the mouse study, compared to our human trial, we observed fewer taxa, and fewer genera differences such as the higher relative abundance of *Akkermansia*, *Bilophila*, *and Alloprevotella* in the HFVK group than in the HF group (Additional file 1: Fig.S5E-S5F). Although the taxa *Akkemansiaceae* and *Desulfovibrionaceae* were not dominant families in our previous human trial, the functional enrichment of the MK-7-regulated microbiota in the mouse model still exhibited a similar enhancement of the capacity for energy metabolism, amino acid metabolism, and protein glycosylation reactions compared to the HF group (Additional file 1: Fig.S5G-S5H). It should be noticed that the genera *Flexilinea* (*P* = 0.031) and *Escherichia-Shigella* (*P* = 0.004) were significantly increased in the HF group, and the genera *Lachnospiraceae_FCS020_group* (*P* = 0.006) and *Lachnospiraceae_AC2044_group* (*P* = 0.017) were significantly decreased in the HF group when compared to the NC group. These four genera were restored after the MK-7 supplementation (all *P* < 0.05, Additional file 1: Fig.S6A). *Flexilinea* belongs to the methanogens, which have been reported to be positively correlated with the increase of blood glucose and blood lipid parameters [33]; *Escherichia-Shigella* are the potentially pathogenic bacteria which have been correlated with the dysbiosis of microbiota [34]; and *Lachnospiraceae* are the family subset of the phylum *Firmicutes* which are known to be responsible for the production of SCFA [35].

### The MK-7-regulated gut microbiota promoted divergent functional alterations in the colon, liver, and pancreas tissue

**Fig. 5:**
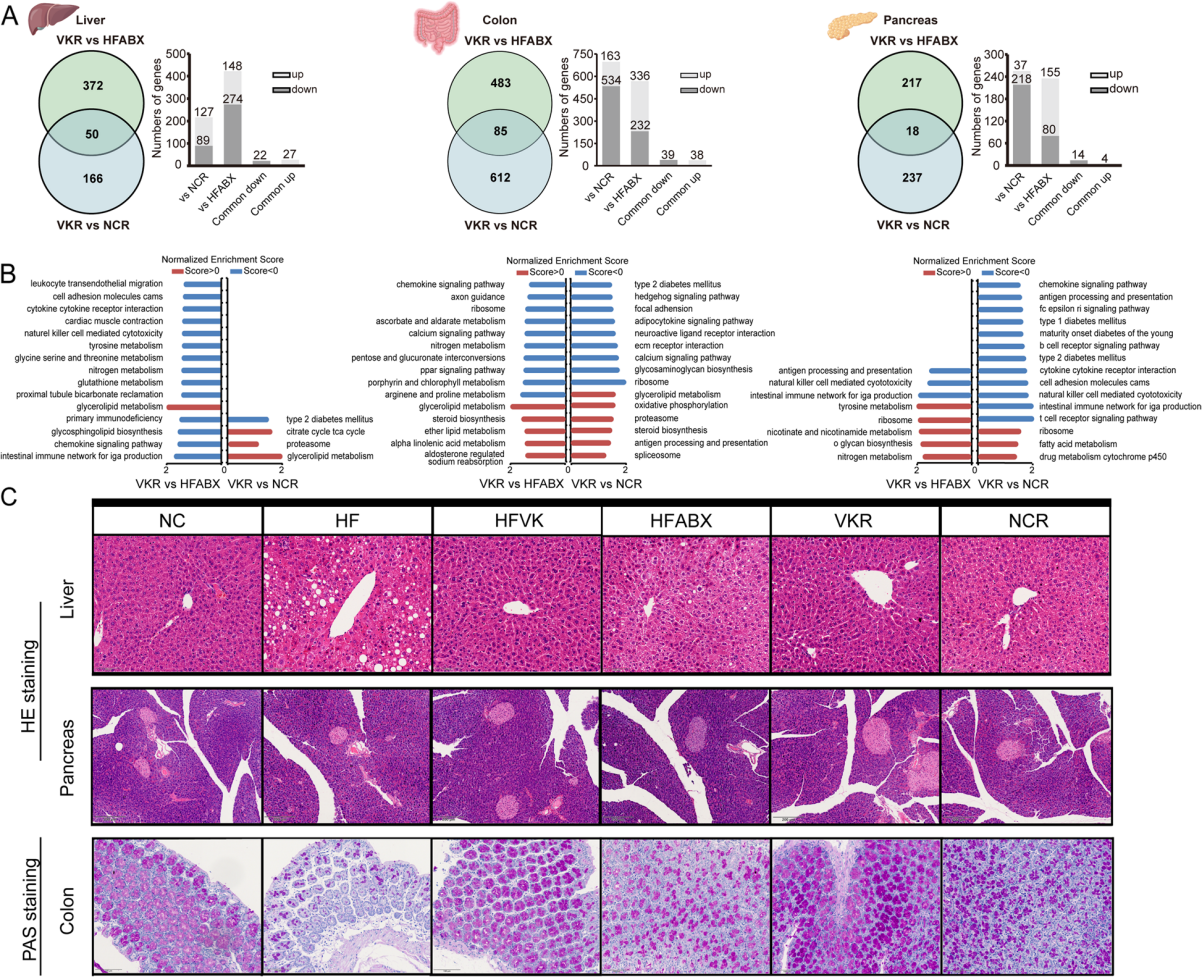
Gene set enrichment analysis and pathologic tissue section revealed potential ability of MK-7-regulated microbiota on modulating host energy metabolism and immunoinflammation. **A** Overview of the difference of gene expression (*P* < 0.05 and │log2fold change│ > 1) in liver, colon and pancreas tissue between the VKR group and HFABX or NCR group. **B** Top 15 significantly altered KEGG pathways (*P* < 0.05) caused by MK-7-regulated microbiota under whole gene set enrichment analysis of the liver, colon, and pancreas tissue, respectively. **C** Liver, pancreas, and colon histopathologic appearance by HE and PAS staining. Scale bars, 100 μm of liver and colon, and 200 μm of pancreas

Due to the alterations in the gut microbiota after MK-7 intervention, we next detected the expression and functional changes in the transcriptomic profiles of colon, liver, and pancreas tissues in FMT mice. DEG analysis of the three tissues revealed hundreds of DEGs between NCR and VKR as well as between HFABX and VKR, of which the pancreas appeared to be the less affected tissue (Fig. 5A). Compared to HFABX mice (almost sterile, with low microbial activity), 22, 20, and 14 pathways were significantly enriched in the liver, colon, and pancreas, respectively (Additional file 1: Table S3). Focusing on the few top pathways based on the normalized enrichment score (NES) and leading-edge subset genes among the three tissues, most were associated with the upregulation of glycerolipid metabolism, lipid metabolism, steroid biosynthesis, and glycan biosynthesis as well as downregulation of the amino acid metabolism and the immunoinflammatory response (Fig. 5B, Additional file 1: Fig.S6B). Similar results were obtained when comparing between NCR and VKR (Fig. 5B). The MK-7-regulated microbiota seemed to have a stronger effect on the colon and pancreas compared to NCR mice and have a stronger effect on the colon and liver compared to HFABX mice.

Additionally, in the histological evaluation of liver tissue, we observed a decreased trend in steatosis as well as reduced inflammatory cells in VKR mice, which were comparable to those in NCR mice (Fig. 5C). However, no significant pathological changes were observed in HE staining of the pancreatic tissue (Fig. 5C). Furthermore, the reduction in colonic and ileal goblet cell density and normal goblet cells caused by the 60% high-fat diet was partially restored by the transplantation of MK-7-regulated microbiota (Fig. 5D, Additional file 1: Fig.S7A).

### Targeted fecal metabolites, RT‒qPCR, and immunohistochemistry further indicated the specific mechanism of the MK-7-regulated microbiota

**Fig. 6:**
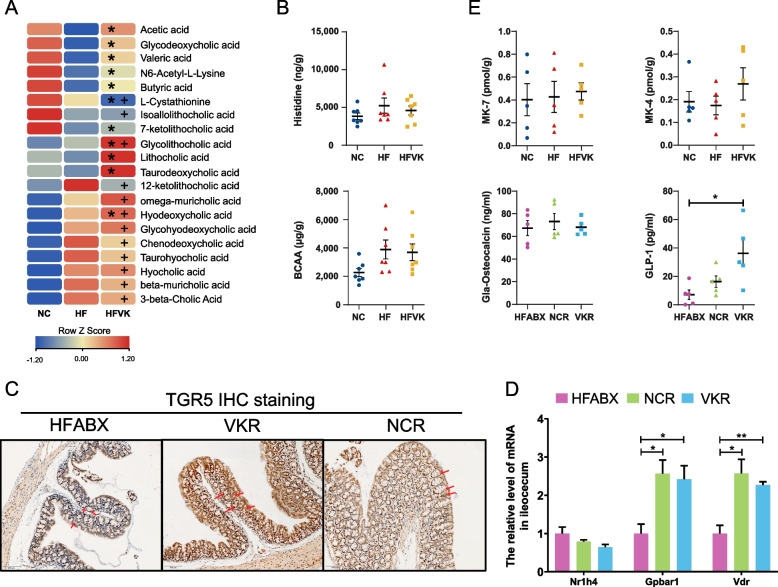
Increased fecal SCFA and SBAs may interpret elevated circulating GLP-1 through activating bile acid receptors. **A** Heatmap showing the top 20 significantly altered fecal metabolites in the HFVK group compared with HF or NC group. The colors changing from blue to red indicate a higher concentration in feces. * represents significant alteration between HFVK and HF group. + represents significant alteration between HFVK and NC group. Significance was determined by Kruskal–Wallis test *P* < 0.05 or VIP > 1. *N* = 7 mice/group. **B** Comparison of fecal histidine and BCAA levels among groups. *N* = 7 mice/group. **C** Expression of TGR5 in colon tissue based on IHC staining. Red arrow indicates the distribution of TGR5 in cytoplasm and plasma membrane. Scale bars, 100 μm. **D** The relative mRNA expression of bile acid receptors in ileocecum (left) and immunohistochemical staining for TGR5 receptor in colon (right). Scale bars, 100 μm. *P < 0.05, ** P < 0.01 by Kruskal–Wallis test with Bonferroni adjustment. *N* = 4 mice/group. **E** Fecal MK-7 and MK-4 levels (up) and circulating gla-osteocalcin and GLP-1 levels (bottom). *P < 0.05 by Kruskal–Wallis test with Bonferroni adjustment. *N* = 5 mice/group

To further understand the gut-derived mechanism by which MK-7 ameliorates inflammation and glycemic homeostasis, we first measured SCFA, bile acid, and amino acid profiles in the feces of the donor group to reconfirm whether MK-7 could influence the effects of the microbiota on these metabolites. Similar to the previous RCT, several SBAs and SCFAs, such as lithocholic acid, 7-ketolithocholic acid, taurodeoxycholic acid, acetic acid, butyric acid, and valeric acid showed an increasing trend in the VK group compared with the HF group (Fig. 6A). We also found that although there was a decreasing trend in short-chain fatty acids compared to the NC group, there was still a significant increase in muricholic acid and deoxycholic acid (Fig. 6A). Additionally, it may be due to the heterogeneity of dietary sources that the fecal amino acids BCAA and histidine exhibited no difference between groups in our mouse model but were found to be significantly decreased in T2DM participants after 6 months of MK-7 intervention (Fig. 6B). On the other hand, consistent with the differences in functional enrichment and fecal metabolites of the VKR group, RT-qPCR showed significantly altered mRNA expression of the bile acid receptor TGR5 (Gpbar1, fold change = 2.42, *P* = 0.036), Vdr (fold change = 2.27, *P* = 0.004), and FXR (Nr1h4, fold change = 0.64, *P* = 0.100), and IHC staining also showed the increased expression of TGR5 in the cytoplasm and plasma membrane of colon tissue in VKR group (Fig. 6C). In addition, the anti-inflammatory interleukins IL2r (fold change = 2.63, *P* = 0.034), IL11 (fold change = 1.97, *P* = 0.031), and IL13 (fold change = 2.15, *P* = 0.004) were observed to be upregulated in the VKR group compared to the HFABX group in ileocecal tissue (Fig. 6D, Additional file 1: Fig.S7B). In visceral adipose tissue, we also found that the mRNA expression of the pro-inflammatory interleukins Il1b (fold change = 0.28, *P* = 0.017), Il6 (fold change = 0.50, *P* = 0.036), and anti-inflammatory interleukin Il13 (fold change = 3.39, *P* < 0.001) was significantly altered (Additional file 1: Fig.S7B). Furthermore, we observed an increased concentration of the circulating incretin GLP-1 in the VKR group compared to the HFABX group and this difference was not found between NCR and HFABX (Fig. 6E).

It should be noted that there are no differences in the concentrations of vitamin K2 (MK-4 and MK-7) in cecal contents and gla-osteocalcin in serum were detected between the donor groups (Fig. 6E), indicating that the improvement induced by FMT is not due to the direct effect of vitamin K2 itself but to the regulation of altered gut microbiota. Taken together, the alterations in the microbial metabolites SBAs and SCFAs, the mRNA expression of bile acid receptors and interleukins in several tissues, and the circulating GLP-1 concentrations with unchanged levels of intestinal vitamin K2 further revealed a potential ability of the MK-7-regulated microbiota to improve host inflammatory status and glycemic homeostasis.

## Discussion

**Fig. 7:**
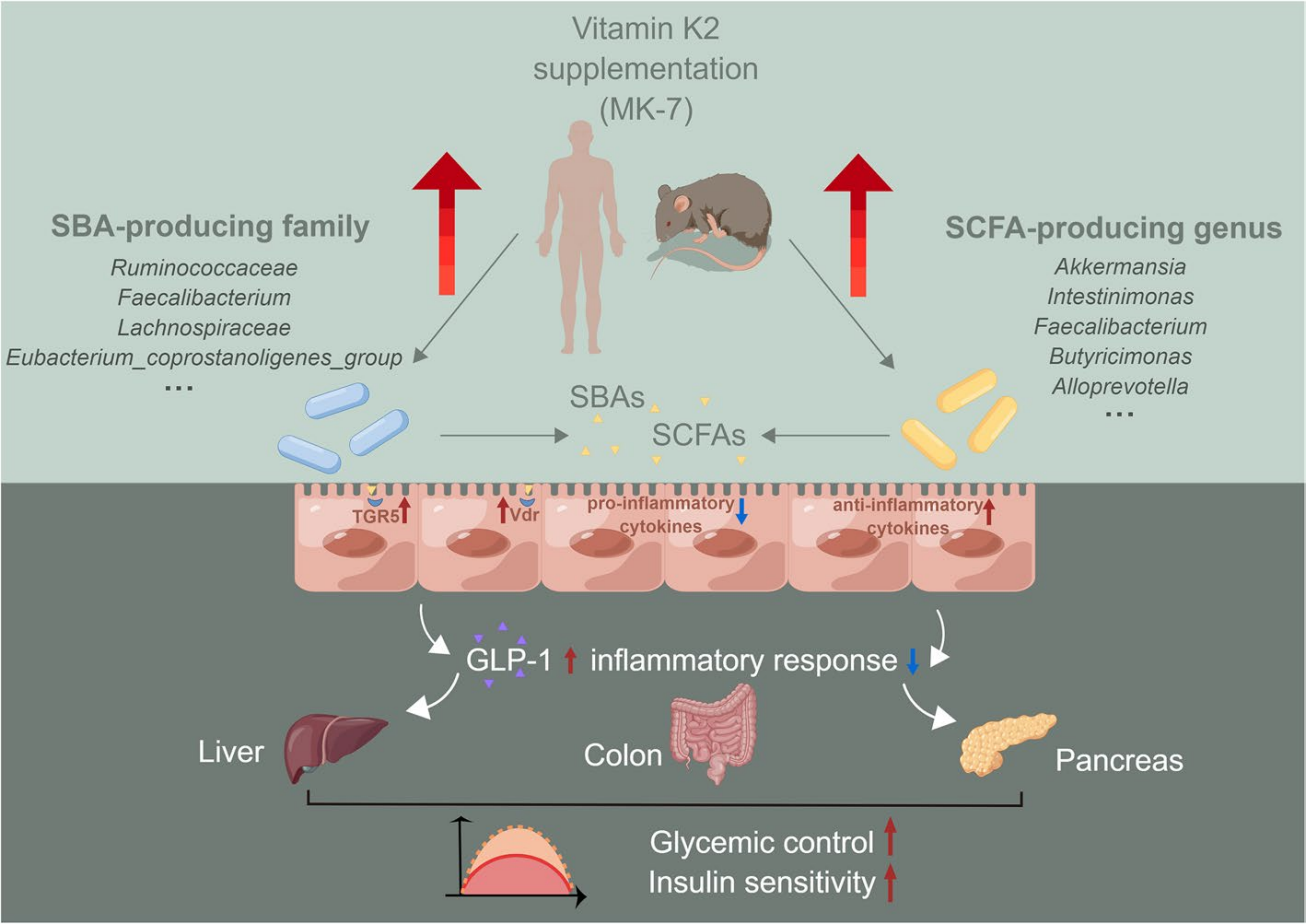
Summary of key findings on how vitamin K2 (MK-7) improves glycemic control and insulin sensitivity in diabetics via gut microbiota and co-metabolites

In this 6-month MK-7 intervention study, we identified a novel mechanism whereby the gut microbiota and its metabolites SBAs and SCFAs are important mediators of the effects of MK-7 on glucose metabolism and insulin sensitivity. Animal studies further confirmed that the improvements in glycemic homeostasis and insulin sensitivity induced by MK-7 can be transferred via FMT through gut-derived mechanisms (Fig. 7).

Interestingly, our present study revealed a significant increase in the abundance of specific gut microbiota constituents, such as *Bacteroidetes* and *Akkermansiaceae*, in human and mouse studies (Additional file 1: Fig.S3C, Fig.S6A), which are known to contribute to the maintenance of adequate vitamin K2 concentrations in both the gut and circulation [36]. From another perspective, *Bacteroidetes*, as an essential commensal bacterium, has been reported to be associated with T2DM and obesity in both animals and humans [37, 38]. Similarly, *Akkermansia* has been proven to be a promising probiotic with multiple health-promoting effects in clinical trials [39]. On the other hand, after MK-7 intervention, the genera *Barnesiella*, *Paraprevotella*, *Turicibacter*, *Anaerofilum*, and *Dielma* were increased in the VK6 group (Fig. 2D), which have been implicated to be involved in the regulation of the immuno-inflammatory response in human and animal studies [40–44]. It should be emphasized that the daily intake of vitamin K2 accounts for only 10–25% of the total vitamin K [45, 46], and a significant proportion of vitamin K2 intake is produced by the gut microbiota [47]. In light of our present findings, the importance of ensuring an adequate intake of exogenous MK-7 for the overall metabolic balance should be emphasized, especially in several metabolic diseases that could lead to a long-term reduction in the VK2-producing microbiota and compromise its important metabolic benefits.

From another perspective, the communication between genera, especially butyrate-producing microbiota within *Firmicutes*, was obviously increased after MK-7 intervention (Fig. 2E, Additional file 1: Fig.S3D). As the decrease of communication in these genera has been reported to be associated with T2DM and obesity [48], Our findings indicate that MK-7 can stabilize the interaction of gut microbiota under the influence of diabetes. In addition, we also confirmed that the improvement of MK-7 nutritional status in the circulation is associated with a lower F/B value (Additional file 1: Fig.S3B), which is considered as a balance indicator of the gut microbiota and has been observed to increase in the type 2 diabetes and obesity [49, 50]. It has been reported that resilience of the gut microbiota after perturbation is a hallmark of health [51], our findings suggested that MK-7 could be treated as a gut stabilizer to promote gut homeostasis and host glycemic status towards a healthy phenotype.

Changes in host phenotype are dependent on the downstream metabolites of the microbiome rather than the composition per se [52]. Similarly, we noted that the effect of MK-7 intervention not only moderately altered the composition of the gut microbiota in T2DM participants and the DIO mouse model, but also drastically increased the concentration of the SBAs and SCFAs in the fecal sample. SCFAs are known to be the downstream metabolites of *Intestinimonas*, *Faecalibacterium*, *Butyricimonas*, *Alloprevotella*, and *Akkermansia* [53–57], and these genera were all found to be increased after MK-7 intervention (Fig. 2D, Additional file 1: Fig.S5E-5F). On the other hand, *Clostridiales*, *Ruminococcaceae, Lachnospiraceae*, and *Eubacterium_coprostanoligenes_group* are capable of bile acid 7alpha-dehydroxylation to produce SBAs, and these taxa were also showed an increasing trend after MK-7 intervention (Fig. 2D, Additional file 1: Fig.S3C, Fig.S5F) [35, 58–61]. To the best of our knowledge, this is the first study to discover the association between MK-7 and increased synthesis of SBAs or SCFAs.

SCFAs and SBAs are highly bioactive metabolites produced by the gut microbiota and play a key role in host energy homeostasis and immune regulation through local and systemic effects on multiple targets [62]. Mechanistically, SCFAs and SBAs have been confirmed to regulate the number and function of colonic Treg cells and B cells, and inflammatory cytokines [63, 64], and to promote GLP-1 secretion by activating TGR5, Vdr, and inhibiting FXR [65, 66]. Two recent trials have also indicated that the metabolic benefits of dietary fiber on insulin resistance and T2D are due to increased microbial secretion of SCFAs, SBAs, and gut-derived GLP-1 [66, 67]. Consistent with the existing evidence, the increase in SBAs and SCFAs after MK-7 intervention is closely related to the activation of bile acid receptors and the increase in circulating GLP-1 concentration (Fig. 6). Therefore, we believed that the production of SBAs and SCFAs represents an important pathway for metabolic benefits of microbiota modulation through MK-7 intervention.

Although the functional enrichment of the 16S data was validated by metabolomic methods, a limitation of the study is the lack of shotgun metagenomic data, which would provide stronger evidence. Second, longer-term follow-up and larger study populations for RCTs are needed to better understand the strength of our conclusions. Finally, although antibiotic treatment offers a more accessible alternative to the germ-free model, the disadvantages are incomplete eradication of the microbiota and the lack of standardized antibiotic regimens.

## Conclusions

In conclusion, combining the existing studies on vitamin K2, our findings revealed MK-7 is a beneficial nutrient for both the host and the gut microbiota. Moreover, the microbiota and its metabolites are key intermediates factors in MK-7 intervention that regulate host glucose metabolism and insulin sensitivity. Given that metabolic diseases can lead to a reduction in VK2-producing microbes, this gut-derived evidence may facilitate the clinical implementation of vitamin K2 as an effective postbiotic for diabetes management.

## Supplementary Information

**Additional file 1: Table S1.** Primer Sequences used for RT-qPCR. **Table S2.** Serum parameters of mice in FMT experiment. **Table S3.** All Significant Pathways in GSEA of KEGG Pathways. **Figure S1.** Schematic workflow of the 6-month study design. **Figure S2.** The growth status of the fecal microbiota after the antibiotic interventionand one week after transplantation. **Figure S3.** Altered microbiota and Firmicutes/Bacteroides ratio, coabundance network, fecal metabolite profilesand microbiota functionafter 6-month MK-7 intervention. **Figure S4.** Altered serum biochemical indicators in donor groupsand receiver groupsafter MK-7 intervention and MK-7-regulated microbiota transplantation. **Figure S5.** Similar alteration of α and β diversity, observed amount of OTUs, significant altered microbiota and functional KEGG pathwaysand relative abundance of Family taxonomy were observed after MK-7 intervention in mouse model. **Figure S6.** Significantly altered microbiota in relation to high-fat diet and restored after supplementation of MK-7and landscape of top 50 leading-edge subset genes in liver, colon and pancreas tissue. **Figure S7.** Histopathological manifestationsand RT-qPCRsuggest the ability of MK-7-regulated microbiota to recover the ileal villi length, goblet cell count and visceral adipose tissue inflammatory response.

## Data Availability

The raw sequence data reported in this paper have been deposited in the Genome Sequence Archive in National Genomics Data Center, China National Center for Bioinformation/Beijing Institute of Genomics, Chinese Academy of Sciences (BioProject:PRJCA015879) that are publicly accessible at https://ngdc.cncb.ac.cn/gsa [68, 69]. The clinical/questionnaire/fecal metabolites data that support the findings of this study are available in Figshare with the identifier 10.6084/m9.figshare.22353985. Other data that support the findings of this study are available on request to the authors.

## Abbreviations

BCAAs: Branched-chain amino acids
DEGs: Differential expression genes
DIO: Diet-induced obesity
FMT: Fecal microbiota transplantation
GLP-1: Glucagon-like peptide-1
LCA: Lithocholic acid
NES: Normalized enrichment score
OGTT: Oral glucose tolerance test
SBAs: Secondary bile acids
SCFAs: Short-chain fatty acids
RCT: Randomized controlled trial
TCHO: Total cholesterol
TG: Triglyceride
T2DM: Type 2 diabetes mellitus
