# Supplementary material for: Vitamin K2 supplementation improves impaired glycemic homeostasis and insulin sensitivity for type 2 diabetes through gut microbiome and fecal metabolites
Source: BMC Med. 2023 May 5;21:174. doi: 10.1186/s12916-023-02880-0 (PMC10163743; doi:10.1186/s12916-023-02880-0)
Supplement: Supplementary file 1 — Additional file 1: Table S1. Primer Sequences used for RT-qPCR. Table S2. Serum parameters of mice in FMT experiment. Table S3. All Significant Pathways in GSEA of KEGG Pathways. Figure S1. Schematic workflow of the 6-month study design. Figure S2. The growth status of the fecal microbiota after the antibiotic interventionand one week after transplantation. Figure S3. Altered microbiota and Firmicutes/Bacteroides ratio, coabundance network, fecal metabolite profilesand microbiota functionafter 6-month MK-7 intervention. Figure S4. Altered serum biochemical indicators in donor groupsand receiver groupsafter MK-7 intervention and MK-7-regulated microbiota transplantation. Figure S5. Similar alteration of α and β diversity, observed amount of OTUs, significant altered microbiota and functional KEGG pathwaysand relative abundance of Family taxonomy were observed after MK-7 intervention in mouse model. Figure S6. Significantly altered microbiota in relation to high-fat diet and restored after supplementation of MK-7and landscape of top 50 leading-edge subset genes in liver, colon and pancreas tissue. Figure S7. Histopathological manifestationsand RT-qPCRsuggest the ability of MK-7-regulated microbiota to recover the ileal villi length, goblet cell count and visceral adipose tissue inflammatory response. [file 12916_2023_2880_MOESM1_ESM.docx]

**Supplementary Method**

The clinical parameter measurements, biological sample collection and biochemical index testing were conducted at the Nutrition and Food Hygiene Teaching and Research Laboratory of Harbin Medical University.

**Process of study design, sample size calculation and fecal collection**

The entire experiment lasted 6 months, and feces were collected at baseline and at the endpoint, while fasting serum samples, anthropometric information, and arteriosclerosis status were collected at the baseline, midpoint and endpoint. Sample size calculation was based on the assumption of a 0.5% difference in Hb1Ac between groups, and finally, 28 participants were needed in each group considering power=0.8, significance=0.05 and a 15% dropout rate. Briefly, 70 participants aged 42-80 years were finally recruited at baseline (NC group=33, VK group=37), of whom 60 subjects reached the study endpoint (NC group=30, VK group=30). It should be noted that given the potential effects of hypoglycemic agents, particularly metformin, on gut microbiota, the participants we included were all taking metformin, with only a small number also taking insulin (NC=4, VK=5, *P*=0.723) or sulfonylureas (NC=5, VK=3, *P*=0.456).

Aside from our intervention, subjects were encouraged to continue their normal routine but adopt an active lifestyle whenever possible. Subjects were recommended not to take any medicines on the collection/examination day and the first two days.

For stool collection^1^, participants were given a fecal sampling kit the day before the sampling day, which included a sampling spoon, three sampling tube, a pair of disposable latex gloves, a mask and a small plastic bag. The kit included an instruction sheet on how to collect feces, emphasizing the importance of collecting feces from the mid and posterior part of the stool and of not getting any liquid on the stool during collection. Each sampling tube collected 300mg stool sample (about the size of 3 soybeans). On the eve of the collection day, each participant was given an insulated bucket containing an appropriate amount of dry ice and instructed to store the fecal samples directly in the bucket on the day of collection. On arrival at the collection site, staff collected the buckets and immediately transferred the sampling tubes to an ultra-low temperature freezer at -80°C.

**Collection of Dietary and Clinical Data**

A food frequency questionnaire based on the China Food Composition Database was used to evaluate the dietary data of the subjects. Fat mass was measured by using a body composition analyzer (IOI 353, Jawon Medical). The ankle-brachial index (ABI) and brachial-ankle pulse wave velocity (baPWV) were measured with an arteriosclerosis diagnostic device (BP-203RPE III, Omron). Fasting blood glucose (FBG), serum triglyceride (TG), total cholesterol (TC), high-density lipoprotein cholesterol (HDL-c), low-density lipoprotein cholesterol (LDL-c), uric acid and creatinine were determined with standard laboratory techniques on an automated biochemical analyzer (7100, Hitachi). Fasting serum insulin (FINS) was determined on an automated clinical immunoassay analyzer (Unicel DxC 800, Beckman Coulter), and whole blood glycosylated hemoglobin (HbA1C) was measured by high-performance liquid chromatography (ADAMS A1c HA-8380, ARKRAY). Serum desphospho-uncarboxylated MGP (dp-ucMGP) was measured with a sandwich (dual-antibody) ELISA kit (Mlbio, Cat# ml060649). The HOMA2 values, including HOMA2-IR, HOMA2-S and HOMA2-β, were calculated by using HOMA2 Calculator v2.2.3 (Diabetes Trials Unit, University of Oxford, http://www.dtu.ox.ac.uk/homa).

**Collection of serum biochemical and fecal data in mouse model**

Mice were housed separately in specific pathogen-free (SPF) conditions with ad libitum access to food and water under a 12-h light/dark cycle at a controlled temperature. Food consumption was monitored for each mouse. Blood samples, liver tissue, pancreas tissue, colon tissue and the contents of the cecum were sampled at termination in week 16. Blood chemistry measurements were quantified using an automatic biochemical analyzer (Modular P800, ROCHE). Serum endotoxin (LPS) levels were quantified using a chromogenic endotoxin quantitation kit (Thermo Scientific™ Pierce™, Cat # A39552S), and serum leptin (Beyotime, Cat# PL696), adiponectin (Beyotime, Cat# PA002), gla-osteocalcin (Takara, Cat#MK127), and glucagon-like peptide-1 (GLP-1, Solarbio, Cat# SEKM-0148) were quantified by using a mouse ELISA kit.

**Evidence of the transplantation**

To ensure the effectiveness of the entire process of transplantation, we inoculated 1,000-fold dilutions of fecal suspensions from the three groups (HFABX, NCR, and VKR) in LB medium after the antibiotic intervention (end of week 12) and one week after transplantation (end of week 13). The growth status of the bacteria was observed 24 hours, 48 hours and 72 hours after inoculation (**Figure S2**).

**Quantitative RT‒PCR of Ileocecal Tissue**

Total RNA was extracted from colon tissue with TRIzol reagent (Invitrogen, Carlsbad, CA), and a High-Capacity cDNA Reverse Transcription Kit (Applied Biosystems, CA, USA) was used to reverse transcribe the RNA into cDNA. Quantitative real-time PCR was performed with SYBR Green PCR Master Mix (Applied Biosystems, CA, USA) on a LightCycler 480 II (Roche) and analyzed using LightCycler 480 software. The oligonucleotide sequences are listed in **Table S1**.

**Pathological Examination and Immunohistochemistry**

Parts of the liver and colon tissues from mice were fixed overnight in 4% paraformaldehyde and embedded in paraffin for histological examination. Routine hematoxylin-eosin staining (H&E staining) was performed for liver and colon sections, and routine periodic acid-Schiff staining (PAS staining) was performed for colon sections. For immunohistochemistry (IHC), colon sections were incubated with anti-GPCR TGR5 antibody (Abcam, Cambridge, MA, USA, Cat# ab72608) overnight at 4°C after antigen retrieval. Then, TGR5 was visualized with horseradish peroxidase (HRP) conjugates using DAB detection. Finally, all sections were counterstained with hematoxylin.

**Bacterial DNA Extraction and Sequencing in the RCT and Mouse Experiments** ^2-6^

Fecal samples from participants and the cecal contents of mice were collected by Novogene (Tianjin, China). Briefly, total genomic DNA from samples was extracted using the CTAB/SDS method, and 16S rRNA genes (V3-V4 region) were amplified using specific primers with barcodes. Finally, sequencing libraries were generated using the NEB Next® Ultra™ DNA Library Prep Kit for Illumina (NEB, USA). Then, the library was sequenced on an Illumina HiSeq platform, and 250 bp paired-end reads were generated. Paired-end reads from the original DNA fragments were merged by using FLASH, and sequences were analyzed using the QIIME software package (Quantitative Insights Into Microbial Ecology). Sequences with ≥97% similarity were assigned to the same OTUs, and the RDP classifier was used to annotate taxonomic information for a representative sequence within each OTU.

**Targeted Metabolomics Profiles**

Targeted metabolomics profiling of fecal samples from humans and mice was performed by Metabo-Profile (Shanghai, China). The fecal samples were prepared and preprocessed as described previously ^7^. All standards were obtained from Sigma‒Aldrich (Stockholm, Sweden). The quality control samples were prepared following the same procedures and were set every 10 fecal samples to ensure reproducibility. Raw data were normalized to account for intergroup differences (Pareto scaling). The value was set to 0 if the measurement fell below the limit of detection.

**Transcriptome Sequencing of Mouse Liver, Pancreas and** **Colon Tissue**

Transcriptome sequencing was performed by Novogene (Tianjin, China). The extracted RNA was subjected to quantification, qualification, library preparation, and sequencing on an Illumina NovaSeq 6000 to generate an end reading of 150 bp pairing. In the final step, clean data were obtained by removing reads containing adapters, reads containing N bases and reads of low quality. All downstream analyses were based on clean data.

**Statistical and bioinformatic analysis details**

Group comparisons of baseline characteristics were performed with independent T tests, and follow-up measurements were compared with an ANCOVA model controlling for the baseline value. Considering the sample size of the study and the Q-Q plot of all clinical characteristics, we finally decided not to transform any variables in the group comparison.

In the RCT, the average sequencing throughput for each sample was approximately 89,361 reads. After quality control and ﬁltering, 64,851 reads per sample on average remained and were used in downstream analyses by the R package ‘microeco’. The observed OTUs and Simpson index were calculated to represent the richness and evenness of alpha diversity, and weighted UniFrac distance was performed to represent beta diversity. Due to the nonnormal distribution of 16S data, genus biomarkers between groups were tested by the Wilcoxon rank-sum test and Lefse analysis (to obtain as much difference information as possible, raw P<0.05 or LDA score>3 was set as the threshold). A coabundance network was built for the samples using Spearman correlations between genera; only the significant correlations (P<0.05) larger than 0.6 or smaller than -0.6 were used for network construction, and visualization of the network was performed in Gephi version 0.9.2. The ‘Tax4fun’ R package was used to annotate the functions of genus biomarkers through the Kyoto Encyclopedia of Genes and Genomes database (KEGG). Different KEGG pathways were identified using the Wilcoxon rank-sum test (*P*<0.05). Similarly, the differences in fecal metabolites were evaluated by using the Wilcoxon rank-sum test and variable importance for the projection score (VIP) (raw P<0.05 or VIP score>1 as the threshold). Partial Spearman correlations revealed the relations between clinical parameters and genera or metabolites at termination by adjusting the corresponding baseline value.

In animal studies, similar analytic methods and screening threshold were used to explore the differences in 16S data (Kruskal-Wallis test and Lefse analysis) and fecal metabolites (Kruskal-Wallis test and VIP) among groups. In brief, 81,265 raw reads and 68,533 effective tags per sample were averaged, and the effective tags were used to perform the 16S downstream analysis. In transcriptome analysis of liver, colon and pancreas tissue, differentially expressed genes (DEGs) were selected under the criteria of │log2fold change│>1 and P<0.05 to identify intergroup gene expression differences, and whole transcriptomes were subjected to gene set enrichment analysis (GSEA) to identify intergroup functional differences by using the R packages ‘DESeq2’ and ‘clusterProfiler’. All data are shown as the mean±SEM in figures.

**Reference**

1. Jones J, Reinke SN, Ali A, Palmer DJ, Christophersen CT. Fecal sample collection methods and time of day impact microbiome composition and short chain fatty acid concentrations. *Scientific reports* 2021; **11**(1): 13964.

2. Wang Q, Garrity GM, Tiedje JM, Cole JR. Naive Bayesian classifier for rapid assignment of rRNA sequences into the new bacterial taxonomy. *Appl Environ Microbiol* 2007; **73**(16): 5261-7.

3. Edgar RC. UPARSE: highly accurate OTU sequences from microbial amplicon reads. *Nat Methods* 2013; **10**(10): 996-8.

4. Haas BJ, Gevers D, Earl AM, et al. Chimeric 16S rRNA sequence formation and detection in Sanger and 454-pyrosequenced PCR amplicons. *Genome Res* 2011; **21**(3): 494-504.

5. Rognes T, Flouri T, Nichols B, Quince C, Mahe F. VSEARCH: a versatile open source tool for metagenomics. *PeerJ* 2016; **4**: e2584.

6. Bokulich NA, Subramanian S, Faith JJ, et al. Quality-filtering vastly improves diversity estimates from Illumina amplicon sequencing. *Nat Methods* 2013; **10**(1): 57-9.

7. Xie G, Wang L, Chen T, et al. A Metabolite Array Technology for Precision Medicine. *Anal Chem* 2021; **93**(14): 5709-17.


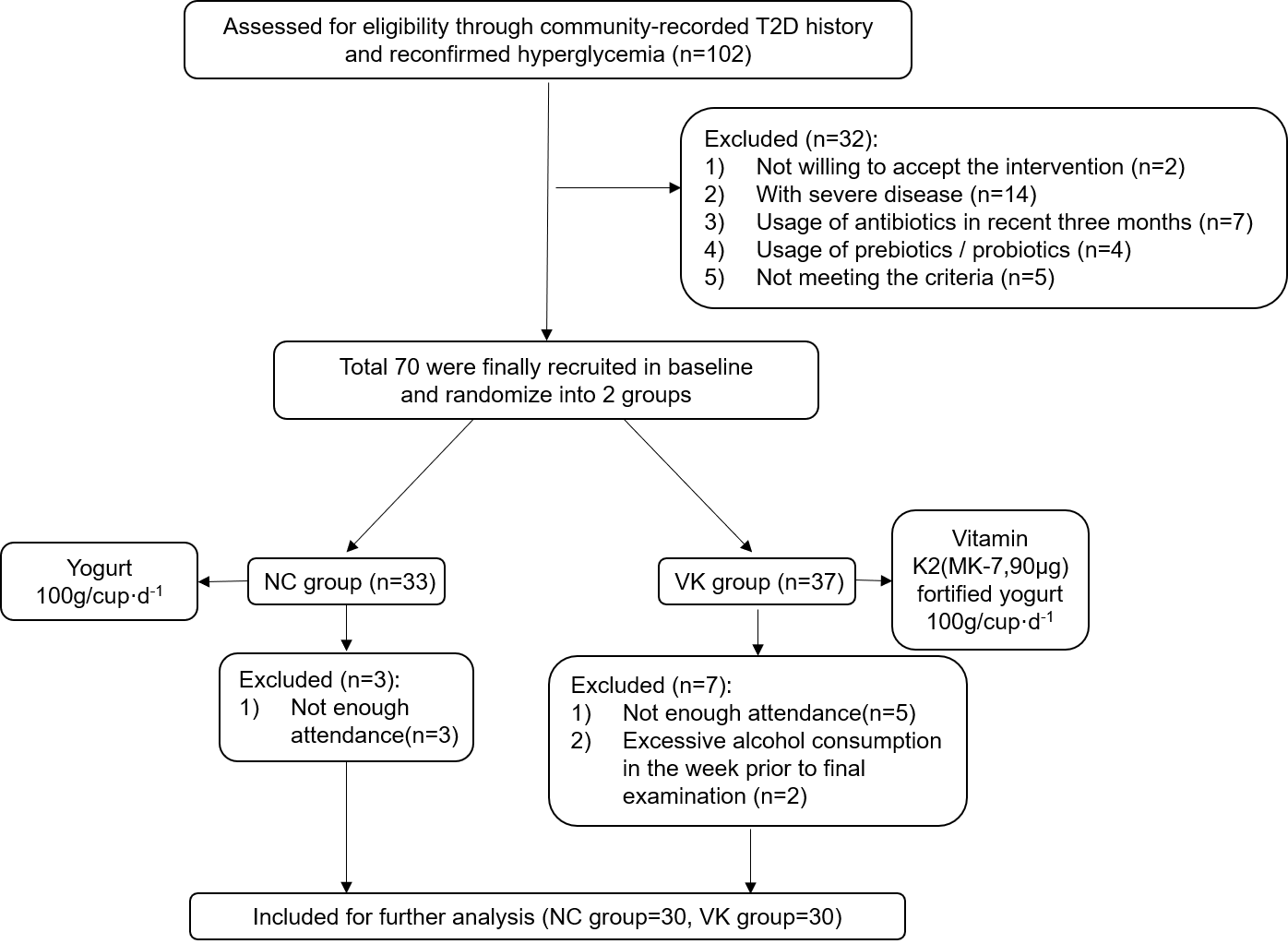


**Figure S1**

**Figure S1.** Schematic workflow of the 6-month study design**.**

**Figure S2**


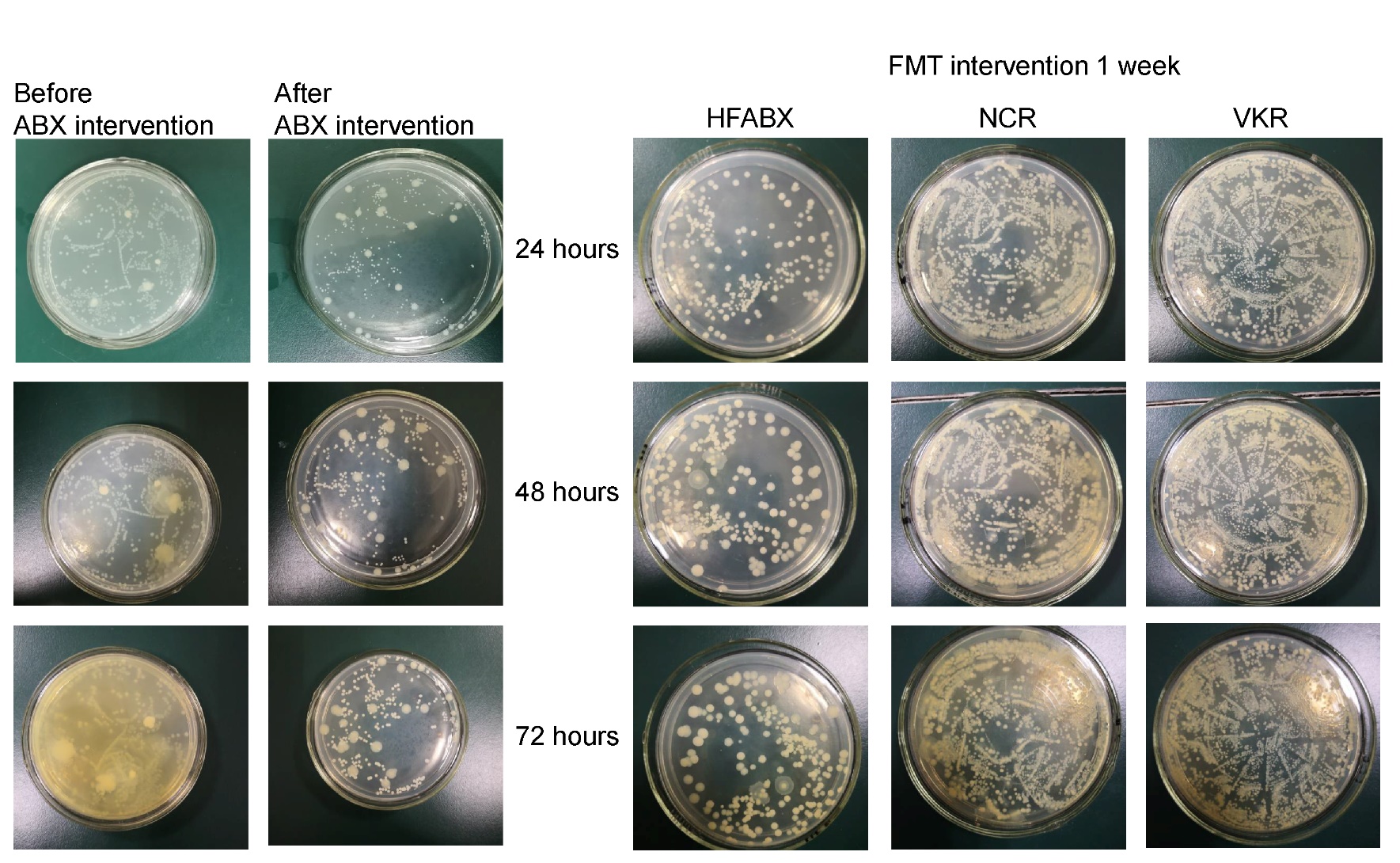


**Figure S2.** The growth status of the fecal microbiota after the antibiotic intervention (end of week 12) and one week after transplantation (end of week 13).

**
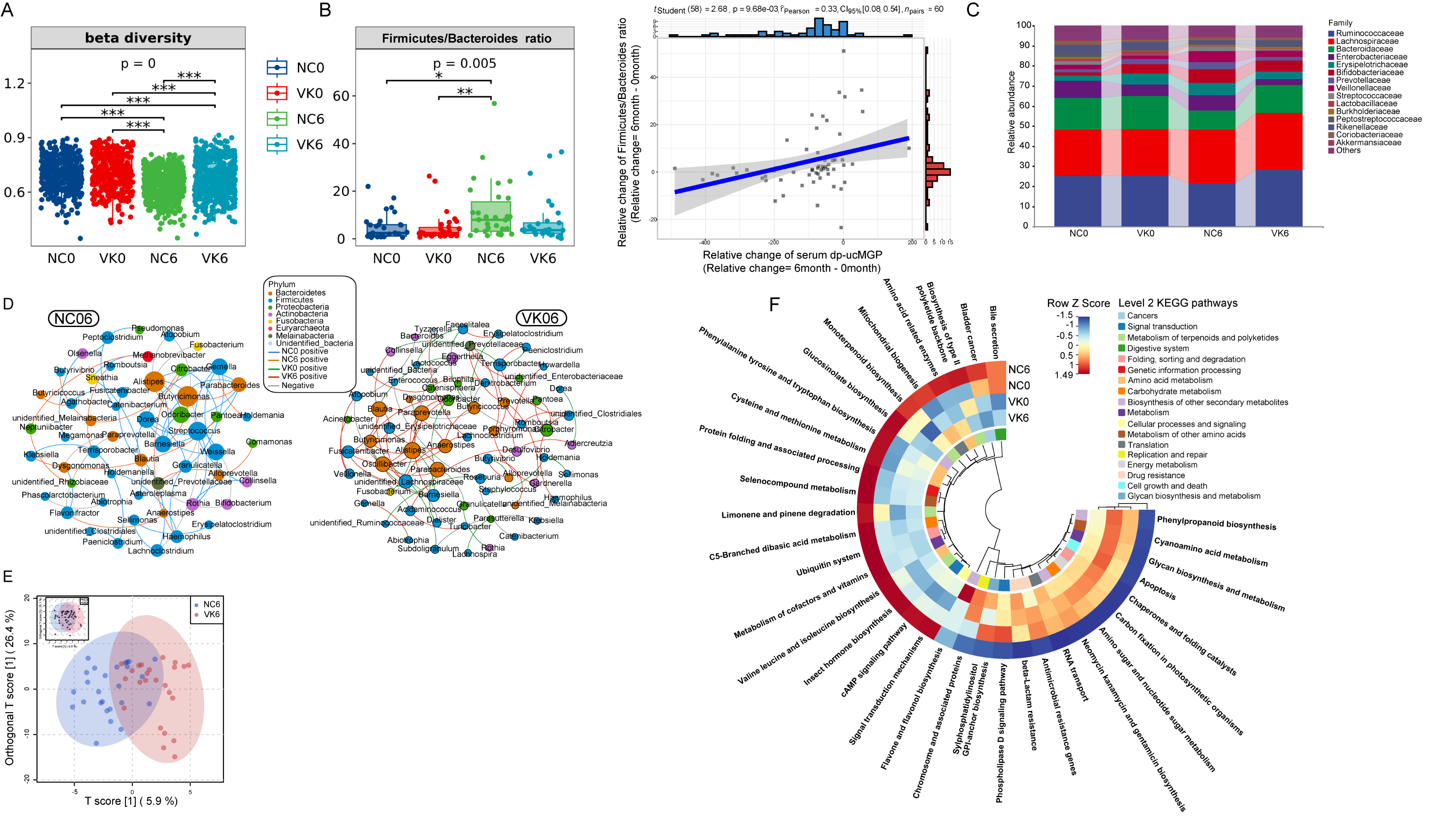
**

**Figure S3**

**Figure S3.** Altered microbiota and Firmicutes/Bacteroides ratio, coabundance network, fecal metabolite profiles and microbiota function after 6-month MK-7 intervention**.**

(A) Wilcox rank sum test of the beta diversity among 4 groups.

(B) Wilcox rank sum test of the *Firmicutes*/*Bacteroides* ratio (left) and Pearson correlation between the relative change of *Firmicutes*/*Bacteroides* ratio (relative change=6 month – 0month) and the relative change of serum dp-ucMGP (relative change=6 month – 0month) (right).

(C) Relative abundance of gut microbiota in Family taxonomy.

(D) Changes of microbiota coabundance network after 6-month study with and without MK-7 intervention. The edges represent significant Spearman correlations of >0.6 or <-0.6 between genera. Each node represents a genus and is colored based on affiliated phylum.

(E) OPLSDA model of fecal metabolites before (top left) and after (main plot) 6-month MK-7 intervention.

(F) Heatmap of significantly altered function of gut microbiota after 6-month MK-7 intervention and showed corresponding baseline status.


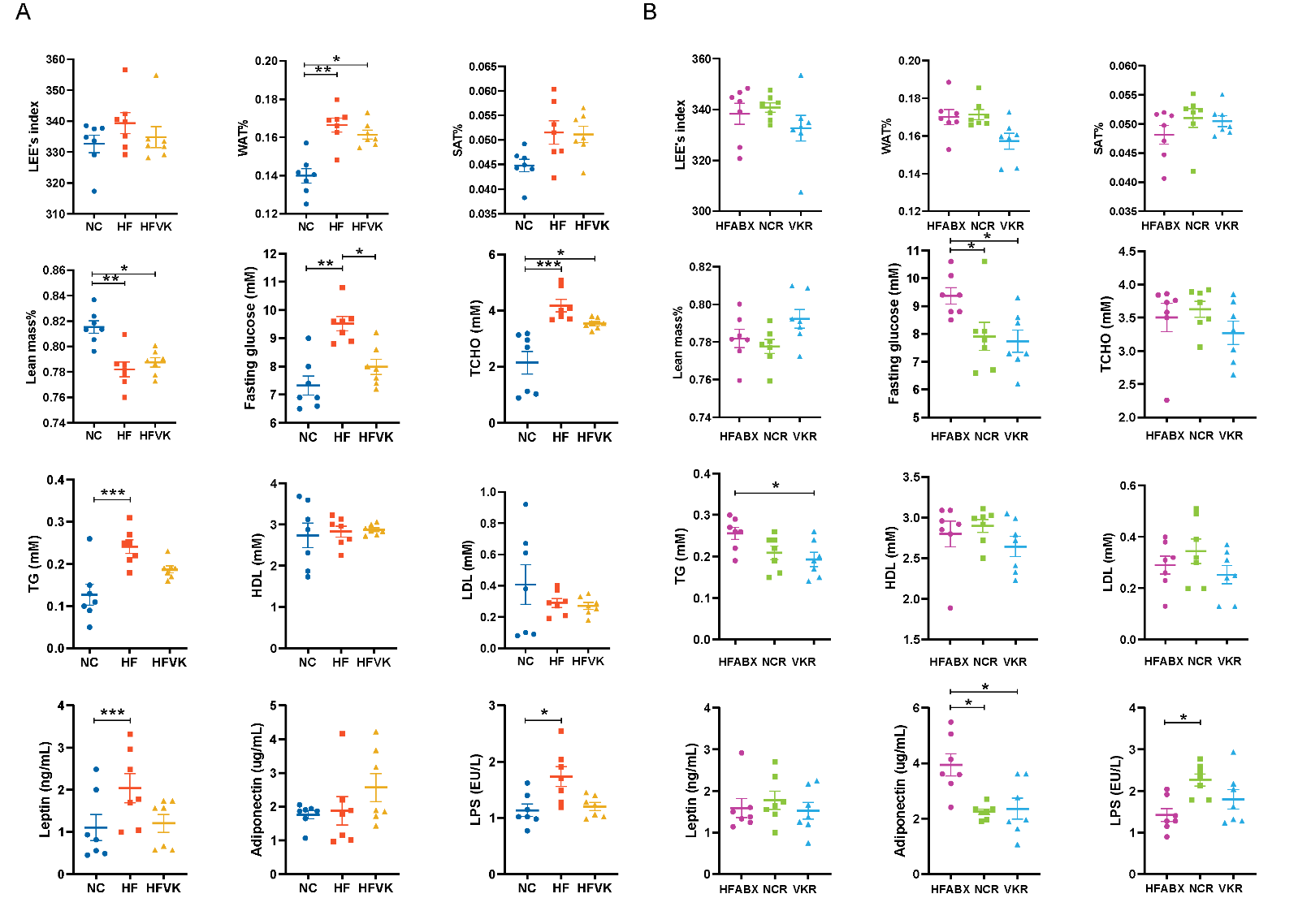


**Figure S4**

**Figure S4.** Altered serum biochemical indicators in donor groups and receiver groups after MK-7 intervention and MK-7-regulated microbiota transplantation.

(A) Altered serum biochemical indicators in donor groups. N=7 mice/group.

(B) Altered serum biochemical indicators in receiver groups. N=7 mice/group.

*P <0.05, ** P<0.01, ***P<0.001 by Kruskal-Wallis test with Bonferroni adjustment.


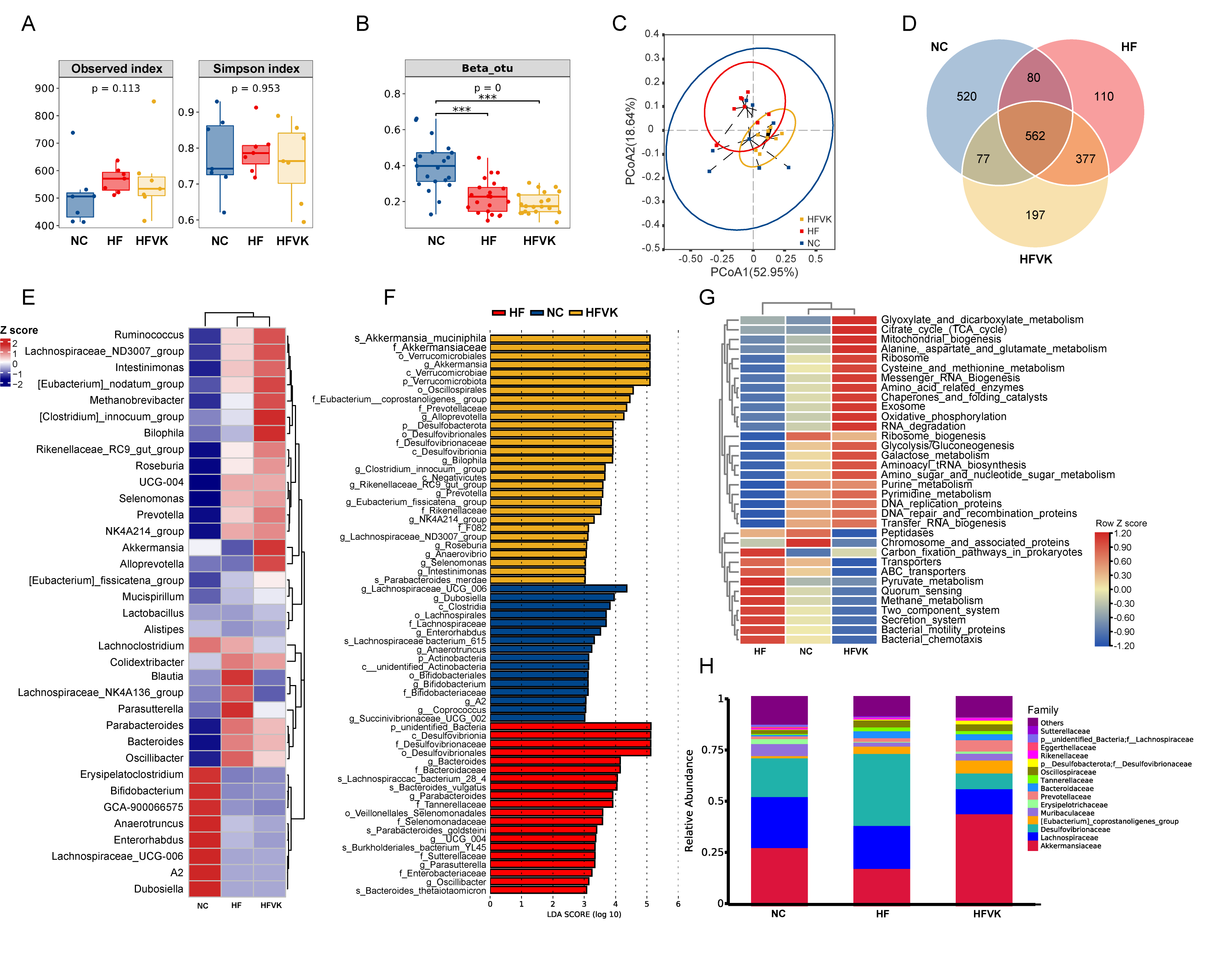


**Figure S5**

**Figure S5.** Similar alteration of gut microbiota was observed after MK-7 intervention in mouse model.

(A-C) α and β diversity of microbiota after MK-7 intervention when compared with HF group.

(D) Overview of the observed amount of OTUs.

(E-F) Metastats (raw *P*<0.05) and Lefse analysis (LDA>3) showed significant altered microbiota after MK-7 intervention when compared with HF group.

(G) Heatmap of significantly altered function of gut microbiota (raw *P*<0.05) after MK-7 intervention when compared with HF or NC group.

N=7 mice/group. *P <0.05, ** P<0.01, ***P<0.001 by Kruskal-Wallis test with Bonferroni adjustment.

(H) Relative abundance of gut microbiota in Family taxonomy.


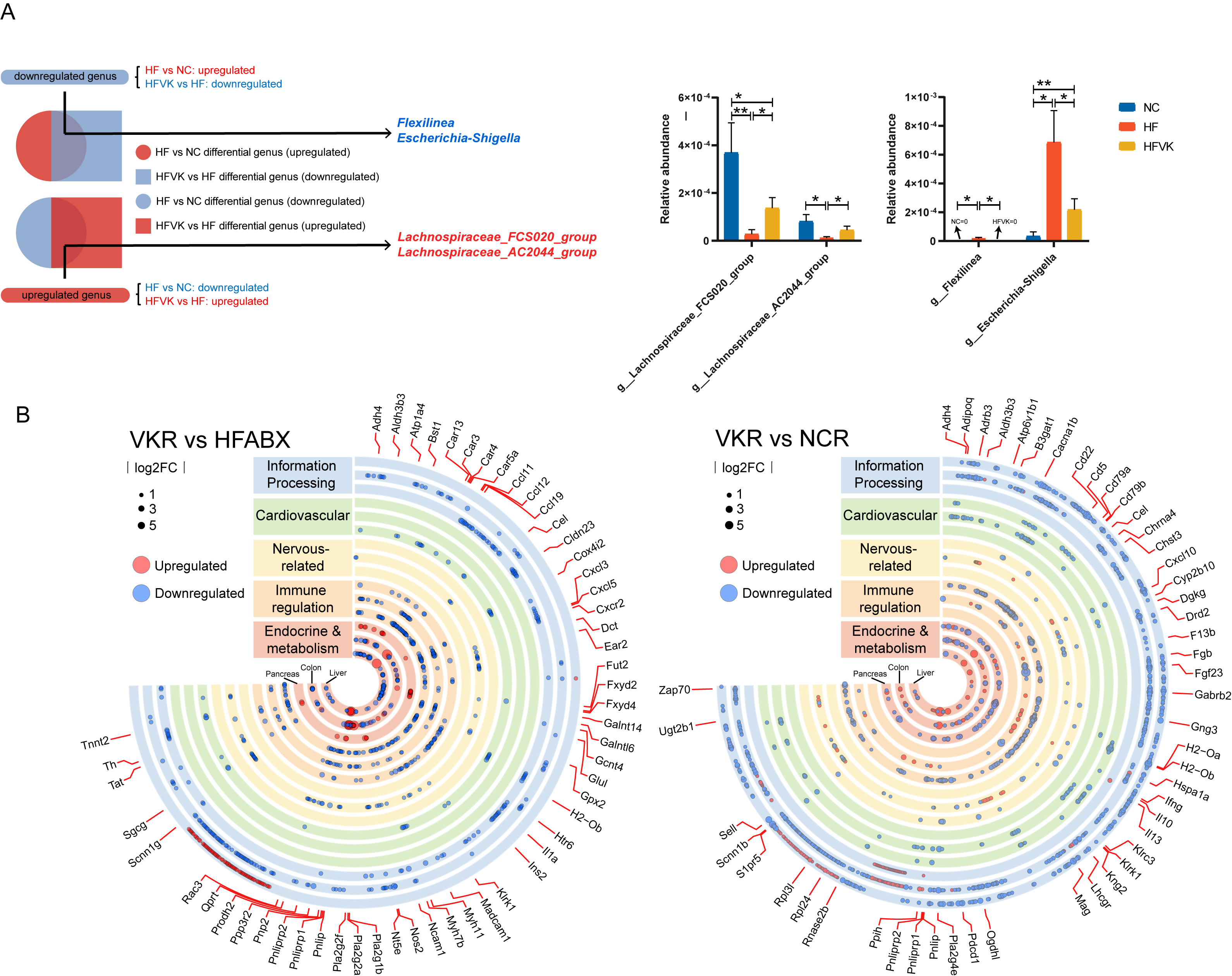


**Figure S6**

**Figure S6.** Shifted microbiota showed the potential ability on modulating host energy metabolism and immunoinflammation.

(A) Metastats (raw *P*<0.05) showed significantly altered microbiota in relation to high-fat diet (HF vs NC) and restored after supplementation of MK-7 (HFVK vs HF).

(B) Landscape of top 50 leading-edge subset genes in liver, colon and pancreas tissue. The regulated genes are labeled as circles (red, upregulated; blue, downregulated). The size of circle indicates │log_2_fold change│.

**Figure S7**


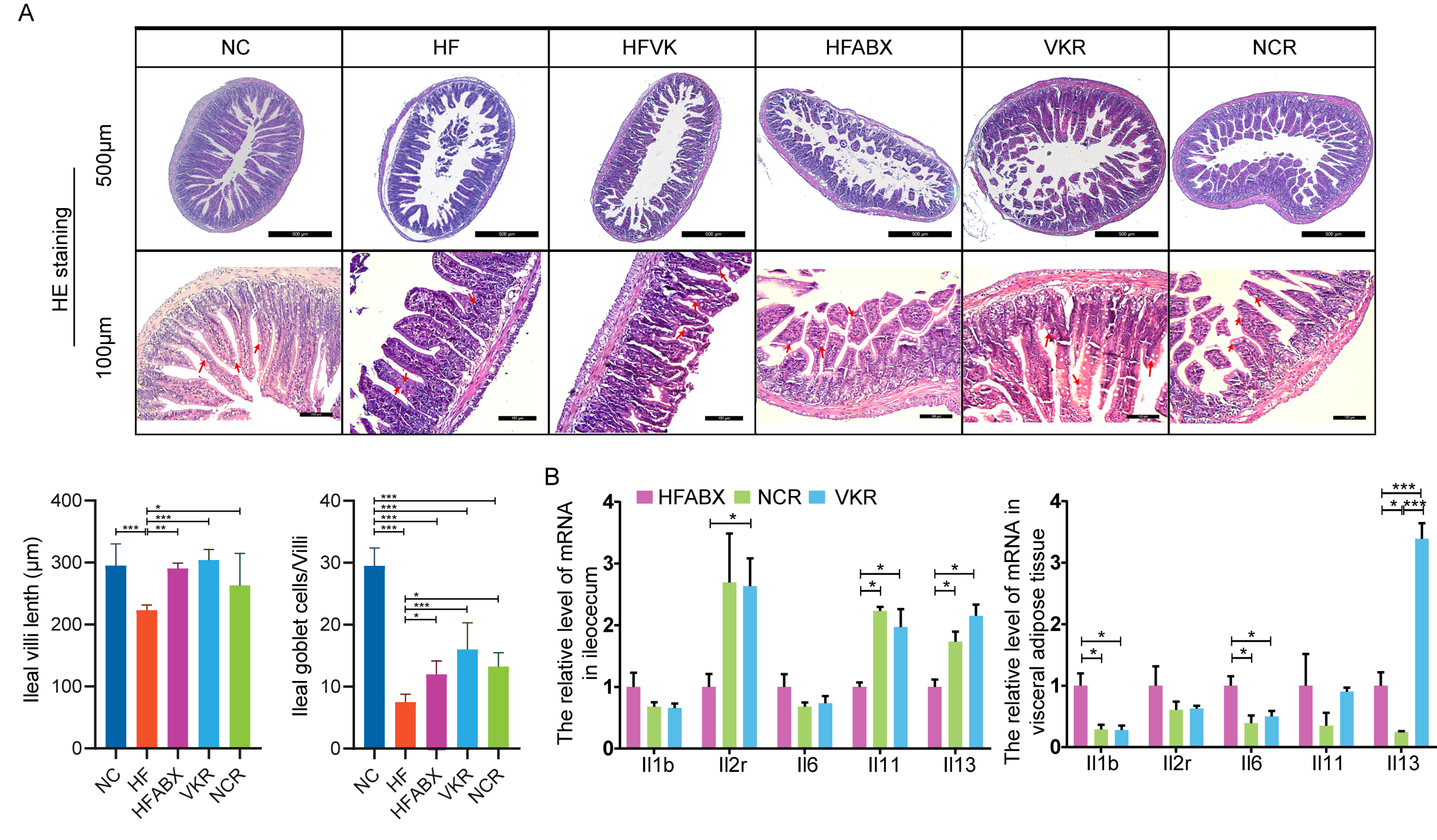


**Figure S7.** Histopathological manifestations and RT-qPCR suggest the ability of MK-7-regulated microbiota to recover the ileal villi length, goblet cell count and visceral adipose tissue inflammatory response.

(A) Ileum histopathologic appearance by HE staining. Scale bars, 500μm (upper) and 100μm (lower). Red arrow indicates the goblet cell.

(B) The relative mRNA expression of interleukins in ileocecum and visceral adipose tissue. N=4 mice/group. *P <0.05, ** P<0.01, ***P<0.001 by Kruskal-Wallis test with Bonferroni adjustment.

| Table S1. Primer Sequences used for RT-qPCR. | | |
| --- | --- | --- |
|  | Forward primer sequences | Reverse primer sequences |
| Gpbar1 | GCCTGGAACTCTGTTATCGCTCATC | GAAGCACTCGTAGACACCTTTGGG |
| Nr1h4 | GCAACCAGTCATGTACAGATTC | TTATTGAAAATCTCCGCCGAAC |
| Vdr | TCAAACTCTGATCTGTACACCC | TGGATGCTGTAACTGACAAGAT |
| Il1b | CACTACAGGCTCCGAGATGAACAAC | TGTCGTTGCTTGGTTCTCCTTGTAC |
| Il2r | TACAAGAACGGCACCATCCTAA | TTGCTGCTCCAGGAGTTTCC |
| Il6 | CTCCCAACAGACCTGTCTATAC | CCATTGCACAACTCTTTTCTCA |
| Il11 | CGAGTAGACTTGATGTCCTACC | GAGACATCAAGAGCTGTAAACG |
| Il13 | GTGTCTCTCCCTCTGAACCTTA | GGGGAGTCTGGTCTTGTGTG |

| Table S2. Serum parameters of mice in FMT experiment | | | | | | | | | | | | |
| --- | --- | --- | --- | --- | --- | --- | --- | --- | --- | --- | --- | --- |
| Group | Fasting glucose(mM) | LEES index | TCHO (mM) | TG (mM) | HDL (mM) | LDL (mM) | WAT% | SAT% | Lean mass% | Leptin(ng/mL) | Adiponectin(ug/mL) | LPS(EU) |
| NC | 7.33±0.83 | 332.65±6.93 | 2.14±0.99 | 0.13±0.06 | 2.74±0.73 | 0.41±0.31 | 0.14±0.009 | 0.04±0.003 | 0.82±0.012 | 0.66±0.15 | 1.77±0.31 | 1.14±0.26 |
| HF | 9.51±0.62 | 339.32±8.37 | 4.17±0.53 | 0.24±0.04 | 2.83±0.32 | 0.29±0.07 | 0.17±0.009 | 0.05±0.006 | 0.78±0.014 | 2.28±0.73 | 1.88±1.03 | 1.74±0.44 |
| VK | 7.99±0.65 | 334.82±8.43 | 3.54±0.18 | 0.19±0.02 | 2.87±0.12 | 0.27±0.05 | 0.16±0.006 | 0.05±0.004 | 0.79±0.009 | 1.18±0.45 | 2.57±1.02 | 1.2±0.17 |
| HFABX | 9.37±0.7 | 338.31±10.17 | 3.5±0.52 | 0.26±0.04 | 2.8±0.39 | 0.29±0.09 | 0.17±0.01 | 0.05±0.004 | 0.78±0.012 | 1.59±0.56 | 3.94±0.98 | 1.42±0.38 |
| NCR | 7.91±1.23 | 340.83±4.56 | 3.63±0.29 | 0.21±0.04 | 2.9±0.2 | 0.34±0.12 | 0.17±0.007 | 0.05±0.004 | 0.78±0.009 | 1.77±0.53 | 2.25±0.25 | 2.27±0.36 |
| VKR | 7.74±0.98 | 332.67±12.49 | 3.27±0.43 | 0.19±0.04 | 2.65±0.3 | 0.25±0.09 | 0.16±0.01 | 0.05±0.002 | 0.79±0.012 | 1.52±0.5 | 2.36±0.92 | 1.8±0.58 |
| Data was shown as Mean±SEM. | | | | | | | | | | | | |

| Table S3. All Significant Pathways in GSEA of KEGG Pathways^a^. | | | | | |
| --- | --- | --- | --- | --- | --- |
| Group comparison | Organ | Pathways | NES^b^ | Raw *P* value | Leading-Edge Subset Genes |
| VKR vs HFABX | Liver | viral_myocarditis | -1.74392 | 0.002 | Myh7b, Myh11, Sgcg, Sgcd, H2-Oa, Cd40lg, Myh15, Ccnd1, Cav1, Cd40, H2-Eb1, Rac2, Itgal, H2-M2, H2-Aa, H2-DMa, H2-DMb1, H2-Ab1, Itgb2 |
|  |  | glycerolipid_metabolism | 2.023189 | 0.003 | Pnlip, Cel, Pnliprp1, Pnliprp2, Gpat3, Dgkh, Gk |
|  |  | chemokine_signaling_pathway | -1.6104 | 0.003 | Cxcl5, Ccl19, Ccl11, Ccl12, Ccl7, Adcy8, Gng4, Gng8, Cxcl13, Pf4, Shc2, Ccr4, Ccl2, Xcl1, Gng13, Cxcr6, Ccr7, Gng10, Tiam1, Tiam2, Pak1, Cxcl10, Hck, Elmo1, Rac2, Cxcr2, Ccr9, Prkcz, Ccl9, Gngt2, Ccr10, Fgr, Prex1, Ccl22, Ccl21a, Ppbp, Xcr1, Ptk2b, Gnb4, Vav1, Ccl3, Cxcl16, Ccl4, Was, Prkcb, Cxcl9, Adcy2, Cxcr3, Cx3cr1, Pik3cd, Pik3cg, Ncf1, Gng3 |
|  |  | intestinal_immune_network_for_iga_production | -1.73673 | 0.003 | Il2, H2-Oa, Cd40lg, Il10, Tnfrsf17, Tnfrsf13b, Cd40, H2-Eb1, Ccr9, Icos, H2-Aa, Ccr10, Il4, H2-DMa, H2-DMb1, Itgb7, H2-Ab1 |
|  |  | allograft_rejection | -1.68372 | 0.005 | Il2, H2-Oa, Cd40lg, Il10, Cd40, H2-Eb1, H2-M2, Fasl, H2-Aa, Tnf, Il4, H2-DMa, H2-DMb1, H2-Ab1 |
|  |  | asthma | -1.69475 | 0.009 | Ccl11, Ear6, Rnase2a, H2-Oa, Cd40lg, Il10, Prg2, Ear10, Cd40, H2-Eb1, H2-Aa, Tnf, Il4, H2-DMa, H2-DMb1, H2-Ab1, Ear2, Ms4a2 |
|  |  | cytokine_cytokine_receptor_interaction | -1.42543 | 0.010 | Cxcl5, Ccl19, Ccl11, Ccl12, Mpl, Ccl7, Il5ra, Il2, Cd40lg, Il10, Ifnlr1, Tnfsf18, Tnfrsf8, Tnfsf11, Il7, Cxcl13, Pf4, Ccr4, Ccl2, Xcl1, Tnfrsf17, Clcf1, Tnfrsf13b, Il25, Gdf5, Lep, Cxcr6, Il21r, Ccr7, Osm, Cd40, Cxcl10, Cxcr2, Tnfrsf18, Ccr9, Il11, Fasl, Ltb, Ccl9, Ccr10, Il2rg, Csf1r, Tnfrsf19, Ccl22, Tnf, Ccl21a, Ppbp, Xcr1, Lifr, Il9r, Il4, Ccl3, Cxcl16, Ccl4, Il12rb2, Cxcl9, Cxcr3, Il2rb, Cx3cr1 |
|  |  | cell_adhesion_molecules_cams | -1.41207 | 0.014 | Cldn23, Ncam1, Cdh15, H2-Oa, Cd40lg, Cldn4, Cldn7, Cldn10, Cldn18, Cd6, Negr1, Cdh1, Cd4, Cldn6, Vcan, Cldn8, Cd40, H2-Eb1, Selplg, Cd34, Itgal, Pdcd1lg2, H2-M2, Icos, H2-Aa, Spn, H2-DMa, H2-DMb1, Itgb7, Sdc3, Cd22, H2-Ab1, Itgb2, Siglec1, Cd274, Cd2, Itgb8, Nectin2, Itga6 |
|  |  | natural_killer_cell_mediated_cytotoxicity | -1.44878 | 0.017 | Shc2, Nfatc2, Klrc1, Ppp3r2, Cd247, Plcg2, Pak1, Zap70, Klrk1, Tyrobp, Rac2, Itgal, Cd244a, H2-M2, Fasl, Lck, Syk, Tnf, Ptk2b, Vav1, Klrc2, Ulbp1, Hcst, Lcp2, Prkcb, Itgb2, Lat, Pik3cd, Pik3cg, Chp2, Cd48, Fcer1g, Tnfsf10, Raet1e, Gzmb, Ppp3cc, Icam2, Fcgr4, Ptpn6, Sh3bp2, Rac3, Nfatc4, H2-T23, Casp3, Pik3r5, Plcg1 |
|  |  | glycosphingolipid_biosynthesis_lacto_and_neolacto_series | -1.6011 | 0.019 | Fut2, Abo, B3galt2, Fut9, B3galt5, St8sia1, Fut7 |
|  |  | primary_immunodeficiency | -1.54103 | 0.020 | Cd40lg, Tnfrsf13b, Cd79a, Cd4, Blnk, Cd40, Zap70, Cd3d, Ciita, Icos, Lck, Il2rg, Cd3e, Btk, Tap1 |
|  |  | glutathione_metabolism | -1.47295 | 0.025 | Gpx2, Gsto2, Ggt1, Gsta1, Gpx5, Gstm2, Gpx7, Pgd, Gpx6, Rrm1, G6pdx, Gstp2, Gstm7 |
|  |  | leishmania_infection | -1.46164 | 0.026 | Nos2, H2-Oa, Il10, Ncf4, Tlr2, Mapk13, H2-Eb1, H2-Aa, Fcgr1, Tnf, Il4, H2-DMa, H2-DMb1, Prkcb, H2-Ab1, Itgb2, Ncf1, Cyba, Stat1, Tgfb2, Fos, Tab1, Fcgr4, Ptpn6, Il1b, Marcksl1 |
|  |  | graft_versus_host_disease | -1.50321 | 0.028 | Il2, H2-Oa, Klrc1, H2-Eb1, H2-M2, Fasl, H2-Aa, Tnf, H2-DMa, H2-DMb1, H2-Ab1, Gzmb, Il1b, Cd86, H2-T23, Cd80, Cd28 |
|  |  | systemic_lupus_erythematosus | -1.47249 | 0.030 | H2-Oa, Cd40lg, Il10, Actn2, C7, Actn3, C1rb, Cd40, H2-Eb1, H2-Aa, C1qa, Fcgr1, C9, C1qc, Tnf, C1qb, H2-DMa, H2-DMb1, C8b, H2-Ab1 |
|  |  | tight_junction | -1.35681 | 0.030 | Cldn23, Myh7b, Myh11, Myl9, Cldn4, Myh15, Actn2, Cldn7, Rab3b, Cldn10, Cldn18, Ctnna3, Actn3, Prkcq, Ppp2r2b, Ppp2r2c, Cldn6, Cldn8, Prkcz, Hcls1, Myl7, Prkcb, Cgn, Mras, Exoc4, Myh14, Epb41l1, Epb41l3 |
|  |  | leukocyte_transendothelial_migration | -1.38705 | 0.033 | Cldn23, Myl9, Cldn4, Actn2, Mmp9, Cldn7, Cldn10, Cldn18, Ctnna3, Actn3, Ncf4, Thy1, Cldn6, Plcg2, Cldn8, Mapk13, Rac2, Itgal, Ezr, Ptk2b, Myl7, Vav1, Prkcb, Txk, Itgb2, Pik3cd, Pik3cg, Cybb, Ncf1, Cyba |
|  |  | cardiac_muscle_contraction | -1.43898 | 0.040 | Tnnt2, Cox4i2, Fxyd2, Cacna2d3, Cox6a2, mt-Co3, Cacna1f, Actc1, Cacng1, Tnnc1, Cox6b2, Atp1b2, Cacng8, Tpm2, Atp1a2, Cox7a1, Uqcr11, Slc8a1, Cox6b1, Cox5b, Cacnb3 |
|  |  | tyrosine_metabolism | -1.44964 | 0.042 | Dct, Il4i1, Aoc3, Aldh3b3, Tyr, Comt, Adh4, Aldh3b1, Tpo, Ddc, Hemk1, Aoc2, Lcmt1, Hpd, Adh5, Fah, Gstz1, Hgd, Maob, Got2, Aox1 |
|  |  | glycine_serine_and_threonine_metabolism | -1.45823 | 0.043 | Aoc3, Phgdh, Dao, Gamt, Gcat, Alas2, Alas1, Gatm, Dmgdh, Sardh, Bhmt, Aoc2, Agxt2, Shmt2, Gnmt, Amt, Maob, Psat1, Gldc, Shmt1, Psph |
|  |  | proximal_tubule_bicarbonate_reclamation | -1.4744 | 0.045 | Car4, Fxyd2, Car2 |
|  |  | nitrogen_metabolism | -1.46969 | 0.046 | Car4, Car12, Car2, Car7, Car6, Car9 |
|  | Colon | glycerolipid_metabolism | 2.146563 | 0.001 | Cel, Pnlip, Pnliprp1, Pnliprp2 |
|  |  | arginine_and_proline_metabolism | -1.64423 | 0.003 | Nos2, Prodh2, Agmat, Arg2, Ckm, Nags, Arg1, Ckb, Nos1 |
|  |  | amyotrophic_lateral_sclerosis_als | -1.56468 | 0.004 | Ppp3r2, Tnf, Grin2c, Grin2a, Prph2, Nos1, Prph |
|  |  | ppar_signaling_pathway | -1.52725 | 0.010 | Olr1, Me1, Fabp1, Slc27a5, Plin1, Slc27a6, Hmgcs2, Apoa2, Fabp2, Apoa5, Fabp6, Acox1, Acox2, Sorbs1, Cyp8b1, Fabp3, Fabp7, Ehhadh, Pltp, Slc27a4, Ppara, Ilk, Pparg, Lpl, Rxra, Cpt2 |
|  |  | calcium_signaling_pathway | -1.48928 | 0.011 | Nos2, Ppp3r2, Htr6, Adora2b, Cckbr, Grin2c, Grin2a, Chrm5, Grm5, Adrb3, Ryr2, P2rx5, Ntsr1, Htr7, Ryr3, Nos1, Tacr2, Mylk, Slc8a2, Bdkrb1, Atp2b4, Adra1a, Grm1, Atp2b3, P2rx2, Plcd4, Ptafr, Adcy8, Pln, Slc8a1, Slc8a3, Cd38, Adcy1, Adrb1, Itpka, Cacna1g, Cacna1c, Mylk3, Cacna1h, Plcb2, Trpc1, Chrm2, Erbb3, Atp2b2, Htr2b, Htr5a, Camk2g, Erbb2, Slc25a4, Chp1, Bst1, Adcy2, Oxtr, Hrh2, Pde1c, Plcz1, Prkaca, Ptk2b, Chrm3, Camk2b, Prkca, Gna11, Pdgfra, P2rx6, Calm3 |
|  |  | ribosome | -1.46181 | 0.012 | Uba52, Rpl39, Rps12, Rpl35, Rpl3l, Fau, Rplp1, Rpl37, Rpl31, Rpl37a, Rpl38, Rpl30, Rpl18, Rps21, Rpl18a, Rps19, Rpl36, Rps5, Rpl35a, Rps27, Rpl23a, Rps20, Rps23, Rps15, Rps7, Rps29, Rpl34, Rplp2, Rpl32, Rpl27a, Rps16, Rps18, Rps27l, Rps17, Rpl36a, Rps9, Rpl11, Rpl10a, Rps3, Rps15a, Rpsa, Rpl28, Rpl22, Rpl26, Rpl12, Rps26, Rps4x, Rpl19, Rpl7, Rps25, Rps28, Rps24, Rpl9 |
|  |  | porphyrin_and_chlorophyll_metabolism | -1.55056 | 0.015 | Ugt1a5, Ugt2b37, Ugt2b38, Ugt1a2, Ugt2b36, Ugt1a1, Cp |
|  |  | focal_adhesion | -1.31264 | 0.017 | Col6a6, Myl7, Actn2, Col1a1, Mylk, Flna, Myl9, Vegfd, Cav3, Flnc, Itga9, Col6a3, Vav2, Col5a1, Itga2, Pdgfc, Itga7, Pak3, Col6a2, Itga2b, Itga11, Thbs2, Tnc, Col1a2, Mylk3, Ccnd1, Fn1, Ilk, Col3a1, Lama1, Itgb5, Lama5, Col6a1, Tnxb, Col11a1, Erbb2, Tnn, Vav3, Lama3, Itga1, Col5a2, Lamc1, Itgav, Col4a2, Itgb3, Itga3, Tln1, Actn1, Shc2, Ctnnb1, Pik3cb, Vegfb, Vcl, Prkca, Itgb1, Col4a1, Pdgfra, Pak6, Rac2, Mapk10, Akt2, Itga5, Lama4, Col4a6, Bcar1, Mapk3, Zyx, Shc3, Src, Pik3r2, Actn3, Thbs3, Pik3cd, Itgb7, Shc1, Elk1, Lamb2, Parvg, Pip5k1c, Grb2, Pxn |
|  |  | axon_guidance | -1.38586 | 0.019 | Ppp3r2, Dcc, Epha7, Epha6, Dpysl5, Unc5d, Slit1, Ablim2, Efna5, Sema3e, Lrrc4c, Slit3, Sema6d, Pak3, Epha3, Efnb3, Rnd1, Sema3a, Ntng1, Chp1, Srgap3, Unc5b, Sema3c, Sema4a, Limk1, Ephb6, Efna4, Itgb1, Pak6, Rac2, L1cam, Epha1, Sema6c, Gnai2, Mapk3, Sema6a, Slit2, Nck2, Sema7a, Sema6b, Sema5b, Plxnc1, Sema4b, Cfl1, Plxna1, Robo1, Plxnb2, Abl1, Efnb1, Ngef, Rhod, Pak4, Ephb1, Rock2, Unc5a, Pak2, Ptk2, Epha2, Rac1, Fyn, Cfl2, Efna1 |
|  |  | steroid_biosynthesis | 1.606484 | 0.020 | Cel, Cyp27b1, Msmo1 |
|  |  | small_cell_lung_cancer | -1.41383 | 0.020 | Nos2, Rarb, Itga2, Itga2b, Ccnd1, Fn1, E2f2, Lama1, Lama5, Ccne1, Rxra, Lama3, Lamc1, Itgav, Col4a2, Itga3, Cks1b, Pik3cb, Itgb1, Col4a1, Apaf1, Akt2, Lama4, Col4a6, Pik3r2, Trp53, Cdkn2b, Nfkb1, Rb1, Traf6, Pik3cd, Pias4, Lamb2 |
|  |  | pentose_and_glucuronate_interconversions | -1.52493 | 0.021 | Ugt1a5, Ugt2b37, Ugt2b38, Ugt1a2, Ugt2b36, Ugt1a1 |
|  |  | chemokine_signaling_pathway | -1.32625 | 0.022 | Cxcr2, Cxcl3, Ccl17, Ccr2, Ccr3, Ccl2, Ccr4, Ppbp, Cxcl1, Cxcl9, Cxcl5, Fgr, Cxcl14, Ccl4, Adcy8, Vav2, Ccr9, Adcy1, Cxcl11, Prex1, Hck, Adcy5, Ccl9, Gng2, Plcb2, Pf4, Gng11, Was, Gng3, Ccl5, Ccr8, Vav3, Cxcl10, Adcy2, Elmo1, Shc2, Gng13, Grk3, Prkaca, Pik3cb, Gng10, Ptk2b, Cxcr3, Gng4, Rac2, Cx3cr1, Arrb1, Akt2, Cxcr6, Bcar1, Prkcz, Jak3, Gnai2, Mapk3, Plcb3, Shc3, Stat3, Cxcl16, Pik3r2, Gngt1, Arrb2, Nfkb1, Ccr1, Pik3cd, Lyn, Gng5, Shc1, Tiam2, Foxo3, Gsk3a, Grb2, Ccl22, Pxn, Jak2, Ccl25, Ccr5, Ncf1, Plcb4, Rock2, Adcy7, Gnb1, Grk5, Csk, Stat5b, Ptk2, Adcy3, Adcy9, Ccl3, Rac1, Sos2 |
|  |  | regulation_of_actin_cytoskeleton | -1.31102 | 0.023 | Myl7, Actn2, Itgad, Chrm5, Mylk, Fgf14, F2, Bdkrb1, Myl9, Fgf12, Fgf15, Itga9, Apc2, Fgf13, Myh10, Vav2, Fgf16, Itga2, Pdgfc, Itga7, Pak3, Itga2b, Itgam, Itga11, Mylk3, Fn1, Diaph3, Itgb2, Arhgef4, Enah, Itgb5, Chrm2, Itgax, Itgae, Was, Fgf3, Vav3, Iqgap3, Gna12, Wasf1, Itga1, Itgav, Mras, Itgb3, Chrm4, Pip4k2a, Itga3, Limk1, Actn1, Fgfr2, Rras, Pik3cb, Chrm3, Itgal, Vcl, Itgb1, Pdgfra, Pak6, Rac2, Itga5, Pfn2, Bcar1, Nckap1l, Mapk3, Ssh1, Iqgap2, Pik3r2, Actn3, Fgfr1, Wasf2, Slc9a1, Pik3cd, Itgb7, Fgf2, Cfl1, Git1 |
|  |  | ether_lipid_metabolism | 1.490865 | 0.028 | Pla2g1b, Pla2g2a, Pla2g2f, Pla2g5, Pla2g10, Pla2g2e, Chpt1, Pafah1b3, Enpp6 |
|  |  | nitrogen_metabolism | -1.50393 | 0.037 | Car5a, Car1, Car12, Hal, Car6, Car8, Cps1 |
|  |  | aldosterone_regulated_sodium_reabsorption | 1.458064 | 0.037 | Fxyd4, Atp1a4, Scnn1g, Ins2, Kcnj1, Irs4 |
|  |  | ascorbate_and_aldarate_metabolism | -1.485 | 0.042 | Ugt1a5, Ugt2b37, Ugt2b38, Ugt1a2, Ugt2b36, Ugt1a1 |
|  |  | leishmania_infection | -1.39083 | 0.046 | Nos2, Tnf, Tlr2, C3, Ifng, Il1b, Itgam, Itgb2, H2-DMa, Tgfb3, H2-Eb1, Fcgr1, H2-Ab1, Fcgr4, Itgb1, Mapk3, Ncf4, Tgfb1, Tab2, Nfkb1, H2-Aa, Traf6, Cyba, H2-DMb1, Irak4, Elk1, Tgfb2, Tab1, Jak2 |
|  |  | alpha_linolenic_acid_metabolism | 1.480926 | 0.049 | Pla2g1b, Pla2g2a, Pla2g2f, Pla2g5 |
|  | Pancreas | graft_versus_host_disease | -1.82429 | 0.003 | H2-Ob, Il1a, Gzmb, Tnf, Klrd1, Klrc1, Ifng, Prf1, Il2 |
|  |  | asthma | -1.82241 | 0.002 | H2-Ob, Ear2, Tnf, Cd40, Fcer1a, Prg2, H2-DMb1, Il4, Ccl11, Fcer1g, H2-Ab1, H2-DMa |
|  |  | allograft_rejection | -1.70846 | 0.005 | H2-Ob, Gzmb, Tnf, Cd40, Ifng, Prf1, Il2, Fas, H2-DMb1, Il12b, H2-M3, Il4 |
|  |  | intestinal_immune_network_for_iga_production | -1.69928 | 0.006 | Madcam1, H2-Ob, Il15, Cd40, Ccr10, Itgb7, Il2, Icos, Itga4, Tnfrsf13b, Icosl |
|  |  | leishmania_infection | -1.64084 | 0.006 | H2-Ob, Il1a, Itgam, Tnf, Nos2, Ifng, Itga4, Tlr2, Jun, Ncf4, Ncf2, Fos, H2-DMb1, Il12b, Jak2, Itgb2, Marcksl1, Il4, Elk1, Irak1, Fcgr1 |
|  |  | systemic_lupus_erythematosus | -1.61982 | 0.008 | H2-Ob, Actn3, Tnf, Cd40, Ifng, C4b, Grin2b |
|  |  | autoimmune_thyroid_disease | -1.51108 | 0.029 | H2-Ob, Gzmb, Cd40, Prf1, Il2, Fas, H2-DMb1, H2-M3, Il4 |
|  |  | natural_killer_cell_mediated_cytotoxicity | -1.40633 | 0.014 | Klrk1, Rac3, Gzmb, Tnf, Syk, Klrd1, Klrc1, Ifng, Lcp2, Lck, Prf1, Mill2, Shc4, Nfatc2, Fas, Casp3, Tnfsf10, Icam2, Hcst, Pik3cg, H2-M3, Lat, Itgb2, Itgal |
|  |  | antigen_processing_and_presentation | -1.34899 | 0.049 | H2-Ob, Klrd1, Klrc1, Cd4, Hspa1l, Hspa1a, Lgmn, Hsp90aa1, Hspa1b, H2-DMb1, Ctss, Calr, H2-M3, Hspa5, Pdia3, H2-Ab1, H2-DMa, Ctsb, Ctsl |
|  |  | nitrogen_metabolism | 1.507622 | 0.038 | Car3, Glul, Car13, Car2, Cps1, Car7, Gls |
|  |  | o_glycan_biosynthesis | 1.575932 | 0.011 | Gcnt4, Galnt14, Galntl6, Galnt9, Galnt18, Galnt16, Galnt11, Galnt15 |
|  |  | nicotinate_and_nicotinamide_metabolism | 1.626062 | 0.011 | Nt5e, Pnp2, Qprt, Bst1, Nmnat2, Nnmt |
|  |  | ribosome | 1.652456 | 0.001 | Rpl3l, Rps10, Rpl7a, Rpl30, Rps27, Rpl24, Rpl17, Rps28, Rps27a, Rplp2, Rps29, Rpl23, Rpl21, Rpl36, Fau, Rpl38, Rps12, Rps18, Rps23, Rpl37a, Rps21, Rpl37, Rps16, Rps7, Rpl15, Rps9, Rpl18a, Rpl11, Rpl39, Rps17, Rpl31, Rpl10, Rpl18, Rpl13, Rpl36a, Rpl5, Rpl27, Rpl13a, Rpl22l1, Rpl28, Rps15, Rpl27a, Rpl23a, Rps11, Rpl32, Rpl35a, Rplp1, Rps19, Rpl35, Rps3, Rps6, Rps8, Rpl14, Rpl6, Rpl4, Rps2, Rps5, Rpl22, Rps20, Rps24, Rpl26, Rps15a, Rpl8, Rpl10a, Rpl29, Rplp0, Rpsa |
|  |  | tyrosine_metabolism | 1.698334 | 0.005 | Th, Tat, Adh4, Aldh3b3, Pnmt, Adh7, Aldh1a3 |
| VKR vs NCR | Liver | glycerolipid_metabolism | 1.849077 | 0.002 | Pnliprp2, Pnliprp1, Pnlip, Cel, Dgkg, Mboat2, Dgki, Pnpla3 |
|  |  | proteasome | 1.496151 | 0.023 | Ifng, Psma8, Psmc6, Psmd8, Psma5, Psmd14, Psmc5, Psme2, Psma3, Psme4, Psmb10, Psmc4, Psmb3, Psmd6, Psma2, Psmb1, Psma4, Psme1, Psmd12, Psma7, Psmc1, Psmb2, Psmb5, Psmb8, Psmc2, Psme3, Psmd13, Psma1, Pomp, Psmd4, Psmb4, Psmb7, Psmd7, Psmb9, Psmd2, Psmb6, Psmf1, Psmc3 |
|  |  | citrate_cycle_tca_cycle | 1.513761 | 0.024 | Ogdhl, Fh1, Pck1, Sucla2 |
|  |  | type_ii_diabetes_mellitus | -1.3982 | 0.044 | Adipoq, Slc2a4, Mafa, Tnf, Pklr, Cacna1e, Cacna1g, Slc2a2, Cacna1a, Prkcz, Gck, Pik3cb, Socs1, Pik3r2, Pik3cg |
|  | Colon | ribosome | -2.03105 | 0.002 | Rpl17, Rpl3l, Rpl7a, Rpl39, Rpl38, Rps29, Rps28, Rpl35, Rpl37a, Rps27a, Rps27, Rps23, Rpl36a, Rps12, Rpl23a, Rpl22, Rps18, Rps21, Rpl34, Rps20, Rpl9, Rpl10a, Rps17, Rpl36, Rpl27a, Rpl37, Rps15a, Rpl30, Rpl26, Rps24, Rps5, Rps15, Rpl31, Rps16, Rpl35a, Rpl12, Rps7, Rps25, Rps19, Rpl32, Rpl14, Rps9, Rps3, Rpsa, Rplp1, Rpl7, Rps13, Rpl19, Rps3a1, Rps6, Rpl13, Rpl29, Rplp2, Rps4x, Rpl23, Rpl27, Rpl18a, Rps8, Rpl11, Rpl15, Rps26, Rps11, Rpl28, Rpl5, Rplp0, Rpl8, Rpl18, Rpl10, Uba52, Rpl6, Fau |
|  |  | arrhythmogenic_right_ventricular_cardiomyopathy_arvc | -1.95738 | 0.002 | Cacna1s, Cacng2, Des, Ryr2, Cacnb2, Sgca, Cacng5, Cacng3, Ctnna3, Ctnna2, Cacng1, Itga8, Cacnb4, Cacna1c, Actn2, Cdh2, Cacng4, Itgb8, Cacna2d3, Itga9, Itga5, Cacna2d1, Cacng6, Cacng7, Itga1, Sgcd, Itgb3, Cacna2d4, Cacnb1, Tcf7l1, Sgcb |
|  |  | dilated_cardiomyopathy | -1.90391 | 0.002 | Cacna1s, Cacng2, Tnnt2, Des, Ryr2, Myh6, Cacnb2, Tpm2, Sgca, Pln, Cacng5, Cacng3, Cacng1, Itga8, Tgfb3, Cacnb4, Adcy5, Cacna1c, Tgfb2, Cacng4, Adcy1, Itgb8, Cacna2d3, Itga9, Itga5, Cacna2d1, Cacng6, Cacng7, Itga1, Tnni3, Sgcd, Adrb1, Tpm1, Myl2, Itgb3, Cacna2d4, Cacnb1, Tnnc1, Sgcb, Dmd, Adcy3, Adcy8 |
|  |  | hypertrophic_cardiomyopathy_hcm | -1.82157 | 0.002 | Cacna1s, Cacng2, Tnnt2, Des, Ryr2, Myh6, Cacnb2, Tpm2, Sgca, Cacng5, Cacng3, Cacng1, Itga8, Tgfb3, Cacnb4, Cacna1c, Tgfb2, Cacng4, Itgb8, Cacna2d3, Itga9, Itga5, Cacna2d1, Cacng6, Cacng7, Itga1, Tnni3, Sgcd, Prkab2, Tpm1, Myl2, Itgb3, Cacna2d4, Cacnb1, Tnnc1, Sgcb |
|  |  | glycosaminoglycan_biosynthesis_heparan_sulfate | -1.79205 | 0.002 | B3gat1, Hs6st3, Hs3st2, Ndst4 |
|  |  | calcium_signaling_pathway | -1.76026 | 0.002 | Adrb3, Lhcgr, Cacna1s, Htr6, Slc25a31, Nos1, Grm5, Adra1a, Htr5a, Ryr2, Ntsr1, P2rx5, Chrm2, Pln, Ryr3, Slc8a2, Atp2b4, Chrna7, Bst1, Adra1d, Tacr2, Mylk, Erbb4, Atp2b2, Plcd4, Cacna1c, P2rx2, Cckbr, Cacna1g, Adcy1, Agtr1a, Htr2a, Trpc1, Slc8a3, Drd1, Camk2g, P2rx3, Avpr1b, Adrb1, Htr7, Slc25a4, Cacna1e, Trhr, Drd5, Oxtr, Pde1a, Tnnc1, Ptafr, Avpr1a, Adrb2, P2rx1, Itpr1, Tacr1, Adcy3, Adcy8, Adora2b, Ptger1, Ptger3, Cacna1b, P2rx6, Plce1, Itpkb, Camk2a, Tbxa2r, Nos2, Nos3, Bdkrb1, Pde1b, Plcb4, Cacna1h, Camk4, F2r, Pdgfra, Cacna1d, Prkacb |
|  |  | ecm_receptor_interaction | -1.72224 | 0.002 | Col6a6, Col2a1, Thbs2, Sv2a, Itga8, Gp9, Itgb8, Itga9, Itga5, Sv2b, Gp6, Gp1ba, Itga1, Fn1, Sdc3, Col1a1, Col5a3, Tnxb, Itgb3, Col5a1, Col6a3, Lamb2, Col5a2, Thbs3, Col6a2, Col6a1, Gp5, Lama5, Vwf, Col3a1, Tnr, Col4a2, Lamc1, Col1a2, Tnc, Tnn, Col4a4, Itga11, Col4a1, Thbs4, Comp, Reln, Lama4, Hspg2, Lama2, Vtn, Lama1 |
|  |  | neuroactive_ligand_receptor_interaction | -1.64517 | 0.002 | Drd2, Gabrb2, Adrb3, Lhcgr, Chrna4, Gh, Gabra1, Htr6, Oprm1, Cnr1, Grm5, Adra1a, Grik1, Htr5a, Sstr4, Gabrg3, Taar2, Gabra2, Ntsr1, Prlhr, P2rx5, Chrna3, Chrne, Npy4r, Npy5r, Chrm2, Grik2, Lep, Agtr2, Gabrb3, Grik4, Adcyap1r1, Grm8, Npffr1, Gcgr, Gabrb1, Galr1, Chrna7, Adra2b, Adra1d, S1pr5, Tacr2, Grik3, Galr2, Grm2, Gabrg2, Gpr156, Grm6, Gabrg1, Chrm4, P2rx2, Npbwr1, Ptgdr, Cckbr, Gria4, S1pr3, Npy1r, Chrna5, Npy2r, Agtr1a, Htr2a, Mc4r, Adra2c, Hcrtr1, Gabrr2, Tshb, Gm5771, Drd1, Gria2, Ptgir, Fpr2, P2rx3, Lpar4, Avpr1b, Gabrq, Adrb1, Htr7, Oprd1, Trhr, Drd5, Chrnb3, Oxtr, Gpr83, Grid1, Htr1b, Glrb, Gabbr1, Chrna2, Grin3b, Ptafr, Avpr1a, Pth1r, P2ry4, Grin2b, Adrb2, Gria3, Gabra3, Nmur2, Sctr, P2rx1, Grik5, Adora1, Tacr1, Adora2b, Ptger1 |
|  |  | adipocytokine_signaling_pathway | -1.61927 | 0.003 | Adipoq, G6pc, Lep, Irs4, Slc2a4, Mapk10, Npy, Acsl6, G6pc2, Rxrg, Prkcq, Cpt1c, Prkab2, Camkk1, Akt3, Agrp |
|  |  | focal_adhesion | -1.56316 | 0.002 | Col6a6, Col2a1, Myl9, Pak3, Flnc, Thbs2, Cav3, Flna, Mylk, Mapk10, Itga8, Actn2, Cav1, Itgb8, Itga9, Itga5, Vegfd, Shc2, Itga1, Fn1, Col1a1, Myl2, Col5a3, Cav2, Tnxb, Akt3, Itgb3, Col5a1, Col6a3, Lamb2, Col5a2, Thbs3, Col6a2, Col6a1, Parvb, Shc3, Vegfc, Lama5, Vwf, Pdgfd, Col3a1, Flt4, Tnr, Col4a2, Lamc1, Col1a2, Igf1, Tnc, Pdgfb, Hgf, Tnn, Col4a4, Actn1, Itga11, Col4a1, Thbs4, Ppp1r12a, Comp, Pdgfra, Ilk, Elk1, Reln, Lama4, Lama2, Tln1, Vtn, Rasgrf1, Vcl, Lama1, Prkcb, Rac2, Itgb1, Capn2, Fyn |
|  |  | melanoma | -1.53759 | 0.005 | Fgf23, Fgf5, Fgf14, Fgf13, Fgf16, Fgf22, Fgf15, Fgf2, Fgf18, Fgfr1, Akt3, Cdkn2a, Fgf4, Pdgfd, Fgf10, Igf1, Pdgfb, Hgf, Pdgfra, Fgf11, Mitf, Fgf1, Fgf7 |
|  |  | hedgehog_signaling_pathway | -1.5309 | 0.012 | Lrp2, Wnt10a, Wnt9b, Gas1, Wnt10b, Dhh, Wnt9a, Wnt2, Wnt11, Wnt8b, Stk36, Wnt7b, Gli2, Ptch2, Wnt5b, Gli3, Bmp6, Hhip, Wnt16, Bmp2, Rab23, Wnt6, Wnt5a, Prkacb, Bmp4 |
|  |  | type_ii_diabetes_mellitus | -1.5122 | 0.017 | Adipoq, Irs4, Slc2a4, Mapk10, Mafa, Cacna1c, Cacna1g, Slc2a2, Pdx1, Cacna1e |
|  |  | tight_junction | -1.43339 | 0.009 | Myh1, Myh15, Myh11, Myh6, Cldn6, Myl9, Ppp2r2b, Ctnna3, Myh2, Ctnna2, Magi2, Mpdz, Ppp2r2c, Cldn9, Actn2, Myh3, Prkcq, Myh10, Cldn5, Jam2, Myl2, Cldn19, Akt3, Rab3b, Pard6g, Map3k20, Mras, Amotl1, Gnai1, Cldn11, Jam3 |
|  |  | mapk_signaling_pathway | -1.42317 | 0.004 | Fgf23, Pla2g4e, Fgf5, Cacna1s, Cacng2, Ntrk1, Fgf14, Ngf, Cacnb2, Fgf13, Mapk8ip2, Cacng5, Cacng3, Flnc, Flna, Fgf16, Cacng1, Mapk10, Fgf22, Bdnf, Tgfb3, Cacnb4, Fgf15, Cacna1c, Tgfb2, Mos, Cacna1g, Cacng4, Hspa2, Fgf2, Cacna2d3, Fgf18, Cacna2d1, Mapk8ip1, Cacng6, Mapt, Cacng7, Fgfr1, Hspb1, Map3k12, Rasgrp2, Cacna1e, Pla2g2e, Nfatc4, Akt3, Cacna2d4, Cacnb1, Map3k20, Fgf4, Rps6ka2, Rasgrp4, Mras, Fgf10, Cacna1b, Rps6ka5, Rras, Pdgfb, Dusp14, Gadd45g, Pla2g2c, Pla2g2d, Cacna1h, Dusp3, Pdgfra, Fgf11, Cacna1d, Elk1, Map4k1, Prkacb, Fgf1, Fgf7, Mef2c, Dusp4, Dusp8 |
|  |  | regulation_of_actin_cytoskeleton | -1.39988 | 0.005 | Fgf23, Fgf5, Fgf14, Fgf13, Chrm2, Myl9, Pak3, Enah, Apc2, Mylk, Fgf16, Fgf22, Itga8, Fgf15, Chrm4, Pfn2, Mos, Actn2, Itgb8, Fgf2, Fgf18, Itga9, Itga5, Rdx, Myh10, Itga1, Fn1, Fgfr1, Wasf1, Cfl2, Myl2, Itgb3, Fgf4, Mras, Limk1, Pdgfd, Gsn, Fgf10, Rras, Pdgfb, Arhgef4, Actn1, Itga11, Bdkrb1, Abi2, Msn, Ppp1r12a, Was, F2r, Pdgfra, Fgf11, Fgf1, Fgf7, Vcl, Pip4k2b, Fgfr3, Rac2, Brk1, Itgb1, Ssh1, Pip4k2a |
|  |  | vascular_smooth_muscle_contraction | -1.36811 | 0.026 | Pla2g4e, Cacna1s, Adra1a, Actg2, Myh11, Kcnmb2, Acta2, Myl9, Cald1, Ppp1r12b, Adra1d, Mylk, Kcnmb1, Adcy5, Cacna1c, Adcy1, Agtr1a, Gucy1a1, Prkcq, Prkg1, Npr2, Ppp1r14a, Gucy1b1, Ptgir, Avpr1b, Myl6b, Pla2g2e, Kcnmb3, Npr1, Avpr1a, Itpr1, Adcy3, Adcy8, Adora2b |
|  |  | complement_and_coagulation_cascades | -1.36234 | 0.046 | Fgb, Kng2, F13b, A2m, Cfd, C8b, F10, Klkb1, F9, F7, Serpina1e, Serpina1b, Fga, F8, C3, Plat, Fgg, Serpind1, F13a1, C7, Serpinc1, Cd46, Cfh, Cfi, Vwf, C4bp, Cr2, Tfpi, Serping1, Masp1, Pros1 |
|  |  | cardiac_muscle_contraction | -1.35601 | 0.049 | Cacna1s, Cacng2, Tnnt2, Ryr2, Myh6, Cacnb2, Tpm2, Cacng5, Cacng3, Cacng1, Cacnb4, Cacna1c, Atp1a2, Atp1a3, Cacng4, Cacna2d3, Atp1b2, Cacna2d1, Cacng6, Cacng7, Tnni3, Tpm1, Myl2 |
|  |  | axon_guidance | -1.30514 | 0.044 | Lrrc4c, Dpysl5, Epha5, Slit1, Efna5, Epha8, Epha7, Pak3, Dcc, Sema3e, Ephb6, Unc5c, Sema3a, Ntng1, Slit3, Ablim3, Epha3, Cfl2, Sema3d, Efna2, Nfatc4, Ntn1, Sema4f, Cxcr4, Efnb3, Limk1, Sema3g, Gnai1, Plxna3, Robo1, Sema6c, Ablim2, Sema4c, Plxnb1, Srgap1, Slit2, Srgap2, Rnd1, Rac2, Sema5b, Itgb1, Fyn, Plxnc1, Epha4 |
|  |  | parkinsons_disease | 1.274129 | 0.045 | Uqcrq, Uqcr11, Ndufs6, Ndufb6, Cycs, Cox5b, Ndufb8, Ndufa4, Cox4i1, Ndufb7, mt-Atp6, Ndufv2, Cox6b1, Atp5h, Atp5o, Sdhb, Cox5a, Ndufs8, Th, Atp5g3, Cyc1, Cox6a1, Ndufb10, Atp5g1, mt-Co3, Uba7, Slc18a1, Ndufa8, Atp5d, Ndufs7, Ndufb2, Vdac3, Ndufv3, Ube2l6, Atp5c1, Uqcrfs1, Sdhd, mt-Atp8, Ndufab1, Ndufa9, Atp5g2, Ndufs3, mt-Co1, Ndufs2, Slc25a5, Atp5b, Casp3, mt-Co2, mt-Nd1, Park7, Uqcrc1, Slc18a2, Uqcrc2, Atp5a1, Sdhc, Ndufv1, Ndufa4l2, Htra2 |
|  |  | spliceosome | 1.296457 | 0.038 | Ppih, Hspa1a, Lsm5, Lsm7, Hspa1b, Snrpf, Lsm4, Magohb, Ccdc12, Snrpd3, Sf3b5, Snrpd1, Txnl4a, Lsm6, Lsm3, Prpf38a, Bud31, Lsm8, Hspa1l, Phf5a, Zmat2, Rbm8a, Alyref, Bcas2, Srsf3, Srsf9, Syf2, Snu13, Snrpa1, Snrnp40 |
|  |  | antigen_processing_and_presentation | 1.467486 | 0.022 | Klrc3, Hspa1a, Gm8909, Psme2, Hspa1b, Cd8b1, H2-Q2, H2-Q1, Cd8a, Klrc1, Tap1, H2-T23, H2-Q10, Hspa1l, Psme1, B2m, Klrd1, H2-Q7, H2-D1, Ctss |
|  |  | allograft_rejection | 1.470717 | 0.039 | Il10, Gm8909, Il5, Il12b, H2-Q2, Prf1, Fasl, H2-Q1, Il2, Gzmb, H2-T23, H2-Q10, Cd40lg, H2-Q7, H2-D1 |
|  |  | steroid_biosynthesis | 1.513537 | 0.043 | Cel, Soat2, Msmo1, Fdft1, Cyp27b1, Tm7sf2, Cyp51, Dhcr24 |
|  |  | proteasome | 1.566352 | 0.021 | Psme2, Pomp, Psmb3, Psma7, Psmb8, Psmb6, Psmb4, Psma6, Psmb9, Psmb10, Psma2, Psmb5, Psma5, Psmd13, Psmb7, Psmd8, Psma4, Psma8, Psma1, Psme1, Psmb2, Psmd14, Psmd6, Psmc2, Psmc1, Psmc3, Psme4, Psmd3, Psmd12, Psmd11, Psmd2, Psmd7, Psmc4, Psmc6, Psmd4 |
|  |  | asthma | 1.595788 | 0.023 | Il13, Rnase2b, Il10, Rnase2a, Epx, Il5, Ear2, Fcer1a, Cd40lg, Fcer1g |
|  |  | oxidative_phosphorylation | 1.632822 | 0.003 | Atp6v1b1, Uqcrq, Uqcr11, Ndufs6, Ndufb6, Cox5b, Ndufb8, Ndufa4, Cox4i1, Ndufb7, mt-Atp6, Ndufv2, Cox6b1, Atp5h, Atp5o, Sdhb, Atp6v0b, Cox5a, Ndufa11, Ndufs8, Atp5g3, Cyc1, Cox6a1, Ndufb10, Atp5g1, mt-Co3, Ndufa8, Atp5d, Ndufs7, Cox11, Ndufb2, Ndufv3, Atp5c1, Uqcrfs1, Sdhd, mt-Atp8, Ndufab1, Ndufa9, Atp5g2, Atp6v1e1, Ndufs3, Atp4a, mt-Co1, Ndufs2, Ppa2, Atp6v0a2, Atp5b, Atp6v1g1, mt-Co2, mt-Nd1, Uqcrc1, Uqcrc2, Atp5a1, Atp6v1d, Sdhc, Ndufv1 |
|  |  | graft_versus_host_disease | 1.644186 | 0.009 | Il1a, Gm8909, H2-Q2, Prf1, Fasl, H2-Q1, Il2, Gzmb, Il6, Klrc1, H2-T23, Il1b, H2-Q10, Klrd1, H2-Q7, H2-D1, Cd86, Cd80, H2-M2, Fas |
|  |  | glycerolipid_metabolism | 1.645038 | 0.007 | Cel, Pnlip, Pnliprp1, Pnliprp2, Awat2, Gpat3, Agpat2, Plpp2, Dgat2 |
|  | Pancreas | primary_immunodeficiency | -2.03134 | 0.001 | Cd79a, Zap70, Lck, Il7r, Tnfrsf13c, Cd8b1, Cd3d, Cd40, Icos, Cd3e, Ptprc, Aicda, Btk, Cd8a, Cd4, Tnfrsf13b, Tap1, Ciita, Il2rg, Cd40lg |
|  |  | t_cell_receptor_signaling_pathway | -1.9448 | 0.001 | Zap70, Pdcd1, Lck, Prkcq, Cd8b1, Lat, Cd3d, Card11, Cd28, Lcp2, Nfkbie, Rasgrp1, Ctla4, Ifng, Grap2, Mapk11, Icos, Cd3e, Ptprc, Pik3cd, Cd8a, Itk, Cd4, Cd247, Pik3cg |
|  |  | intestinal_immune_network_for_iga_production | -1.92546 | 0.001 | H2-Ob, H2-Oa, Il6, Tnfrsf13c, Cd28, Il15, Itgb7, Cd40, Icos, Itga4, Aicda, Ccr9, Icosl, Ccl28, Tnfrsf13b, Madcam1, H2-DMb1, Cd86, H2-Ab1, Cd40lg |
|  |  | hematopoietic_cell_lineage | -1.84437 | 0.001 | Cd22, Cd5, Il6, Fcer2a, Il7r, Gp9, Cd8b1, Itgam, Il1a, Il9r, Cd2, Cd3d, Flt3l, Gp1ba, Cd55, Csf3r, Itga2b, Ms4a1, Cd3e, Itga4, Csf3, Il5ra, Cd8a, Itga2, Cd4, Flt3, Il2ra, Cd37, Thpo, Itgb3, Fcgr1, Cd44, Il1b, Il4, Cd33, Il1r1, H2-Eb1, Cd59b, Cd34, Csf2ra, Il4ra, Cd1d1 |
|  |  | natural_killer_cell_mediated_cytotoxicity | -1.81313 | 0.001 | Zap70, Klrk1, Lck, Syk, Lat, Hcst, Klrd1, Lcp2, Klrc1, Cd244a, H2-M2, Ifng, Mill2, Rac2, Gzmb, Fcgr4, Pik3cd, Klrc2, Shc4, Fasl, Prf1, Cd247, Prkcb, Tnfsf10, Rac3, Pik3cg, Shc2, H2-Q7 |
|  |  | cell_adhesion_molecules_cams | -1.80077 | 0.001 | Cd22, H2-Ob, H2-Oa, Mag, Sell, Pdcd1, Cntn1, Nlgn3, Cd8b1, Itgam, Cd2, Cd28, Itgb7, Nlgn1, H2-M2, Ctla4, Cntnap2, Cd40, Icos, Spn, Ptprc, Itga4, Cd8a, Cd4, Icosl, Madcam1, H2-DMb1, Ncam1, H2-Q7, Cldn4, Cd86, Vcam1, H2-Ab1, Cldn23, Cd40lg, Pdcd1lg2, Cdh2, Icam1, Nrxn2, H2-M3, Nectin1, L1cam, Itgal, H2-Eb1, Cadm3, Vcan, Cd34, H2-T23, Itga8, Cldn9, Jam3, Cldn6, Cdh4 |
|  |  | systemic_lupus_erythematosus | -1.76598 | 0.001 | H2-Ob, H2-Oa, Cd28, C8b, Ifng, C6, C4b, C2, Cd40, Grin2a, Actn3, Fcgr4, C3, Grin2b, H2-DMb1, Cd86, H2-Ab1, Cd40lg |
|  |  | graft_versus_host_disease | -1.76236 | 0.003 | H2-Ob, H2-Oa, Il6, Il1a, Cd28, Klrd1, Klrc1, H2-M2, Ifng, Gzmb, Fasl, Prf1, H2-DMb1, H2-Q7, Cd86, H2-Ab1 |
|  |  | cytokine_cytokine_receptor_interaction | -1.75767 | 0.001 | Cxcl10, Il6, Il7r, Tnfrsf13c, Osm, Cxcl1, Ccl22, Il1a, Ccl5, Il9r, Relt, Ltb, Cd27, Il15, Flt3l, Lep, Ccl12, Mpl, Ifng, Cxcr6, Tslp, Ccr7, Il21r, Cd40, Csf3r, Tnfrsf25, Csf3, Il5ra, Il2rb, Ccl2, Cxcl9, Ccr9, Tnfrsf14, Fasl, Prlr, Ccr1, Ccl28, Tnfrsf13b, Tnfsf10, Flt3, Il2ra, Bmp2, Ngfr, Tnfrsf1b, Tnfsf14, Tnfrsf4, Cx3cl1, Il2rg, Plekho2, Cd40lg, Tnfsf8, Ccl3, Tnfsf18, Il23a, Ppbp, Ccr4, Inhbc, Tnfrsf11b, Cx3cr1, Il12rb2, Ccr10, Tgfb2, Il10ra, Ctf1, Vegfd, Ccr5, Ccl27a, Inhba, Il1b, Il4, Il1r1, Ccl11, Cxcr3, Lif, Ccl24, Ifnar2, Csf2ra, Tnfsf15 |
|  |  | leishmania_infection | -1.73704 | 0.001 | H2-Ob, H2-Oa, Itgam, Il1a, Ifng, Mapk11, Itga4, Fcgr4, Ncf4, C3, Prkcb, H2-DMb1, H2-Ab1, Nos2, Ncf2, Fos, Fcgr1, Tgfb2, Ptgs2, Marcksl1, Tlr2, Il1b, Il4, Stat1, H2-Eb1, Jak2, Ncf1, Nfkbia, H2-Aa |
|  |  | type_ii_diabetes_mellitus | -1.72408 | 0.001 | Cacna1b, Cacna1e, Adipoq, Hk3, Mafa, Cacna1g, Kcnj11, Mapk10, Pik3cd, Ins2, Abcc8, Pik3cg, Slc2a2 |
|  |  | autoimmune_thyroid_disease | -1.70067 | 0.003 | H2-Ob, H2-Oa, Cd28, H2-M2, Ctla4, Cd40, Gzmb, Tshr, Cga, Fasl, Prf1, H2-DMb1, H2-Q7, Cd86, H2-Ab1, Cd40lg |
|  |  | taste_transduction | -1.68754 | 0.004 | Scnn1b, Cacna1b, Gng3, Tas1r1, Trpm5 |
|  |  | b_cell_receptor_signaling_pathway | -1.67198 | 0.001 | Cd22, Cd79b, Cd79a, Syk, Card11, Nfkbie, Rac2, Btk, Pik3cd, Prkcb, Rac3, Pik3cg |
|  |  | viral_myocarditis | -1.62563 | 0.003 | H2-Ob, H2-Oa, Cd28, H2-M2, Cd40, Cd55, Rac2, Myh6, Myh7b, Prf1, Rac3, H2-DMb1, H2-Q7, Cd86, H2-Ab1, Cd40lg, Icam1, H2-M3, Casp9, Itgal, Myh11, H2-Eb1, H2-T23, Cav1, Myh10, H2-Aa |
|  |  | maturity_onset_diabetes_of_the_young | -1.62504 | 0.010 | Mafa, Neurod1, Neurog3, Nkx6-1, Pax6, Ins2, Iapp, Slc2a2 |
|  |  | type_i_diabetes_mellitus | -1.62315 | 0.006 | H2-Ob, H2-Oa, Il1a, Cd28, H2-M2, Ifng, Gzmb, Fasl, Prf1, Ins2, H2-DMb1, H2-Q7, Cpe, Cd86, H2-Ab1, Ptprn, Gad1, H2-M3, Il1b, H2-Eb1, H2-T23 |
|  |  | fc_epsilon_ri_signaling_pathway | -1.61449 | 0.003 | Syk, Pla2g2d, Lat, Pla2g4a, Lcp2, Mapk11, Mapk10, Rac2, Pla2g2f, Fcer1a, Btk, Pik3cd, Prkcb, Rac3, Pik3cg |
|  |  | antigen_processing_and_presentation | -1.57789 | 0.007 | H2-Ob, H2-Oa, Cd8b1, Klrd1, Klrc1, H2-M2, Klrc2, Cd8a, Cd4, Tap1, H2-DMb1, Hspa1a, H2-Q7, Ciita, Hspa1l, H2-Ab1, Ctss, Cd74, H2-M3, H2-Eb1, H2-T23, B2m, H2-Aa, Hspa2, Tap2, Rfx5, Tapbp, Hsp90aa1, H2-D1, Hspa1b, H2-DMa, Creb1 |
|  |  | allograft_rejection | -1.57585 | 0.013 | H2-Ob, H2-Oa, Cd28, H2-M2, Ifng, Cd40, Gzmb, Fasl, Prf1, H2-DMb1, H2-Q7, Cd86, H2-Ab1, Cd40lg |
|  |  | arrhythmogenic_right_ventricular_cardiomyopathy_arvc | -1.56705 | 0.003 | Ryr2, Ctnna3, Itgb7, Itga2b, Itga4, Cacnb3, Actn3, Lef1, Itga2, Itga10, Cacna2d1, Itga7, Cacng2, Cacna1f, Cdh2, Itgb3, Cacna2d2, Itga11, Itga8, Tcf7l2, Cacna1c |
|  |  | glycosaminoglycan_biosynthesis_chondroitin_sulfate | -1.54561 | 0.015 | Chst3, Chsy3, Chst11, B3gat2, Chst12, Ust, Chst7, Dse, Chsy1, Chst14 |
|  |  | asthma | -1.52961 | 0.017 | H2-Ob, H2-Oa, Cd40, Fcer1a, H2-DMb1, H2-Ab1, Cd40lg, Prg2, Il4, Ccl11, H2-Eb1, Fcer1g, H2-Aa |
|  |  | chemokine_signaling_pathway | -1.52594 | 0.001 | Cxcl10, Gng3, Cxcl1, Ccl22, Ccl5, Dock2, Ccl12, Cxcr6, Ccr7, Rac2, Ccl2, Cxcl9, Pik3cd, Ccr9, Shc4, Itk, Ccr1, Ccl28, Prkcb, Was, Pik3cg, Shc2, Cx3cl1, Ccl3, Gnb3, Ppbp, Ccr4, Adcy5, Adcy7, Cx3cr1, Hck, Ccr10, Grk4, Grk3, Ccr5, Fgr, Ccl27a, Rasgrp2, Stat1, Ccl11, Cxcr3, Jak2, Ccl24, Ncf1, Prex1, Ccl9, Nfkbia |
|  |  | calcium_signaling_pathway | -1.52082 | 0.001 | Cacna1b, Cacna1e, Pde1c, Nos1, Bdkrb1, Ryr2, Hrh1, Pln, Cacna1g, Ryr1, Phkg1, Atp2a1, Tbxa2r, Grin2a, Adrb3, Adra1b, Cacna1h, Avpr1b, Ednra, Hrh2, Prkcb, P2rx5, Cckbr, Atp2a3, Trpc1, Camk4, Adora2b, Adra1d, Tacr1, Cacna1f, Slc8a3, Htr4, Nos2, Slc8a2, Adcy7, Cacna1i, Mylk3, Grin2d, Ltb4r2, Cysltr2, Atp2b2, Grin2c, Adora2a, Grin1, Ptafr, Plcg2, Bdkrb2, Ptger1, Camk2g |
|  |  | prion_diseases | -1.49733 | 0.025 | Il6, Il1a, Ccl5, C8b, C6, Hspa1a, Ncam1, Egr1 |
|  |  | neuroactive_ligand_receptor_interaction | -1.45566 | 0.001 | S1pr5, Gabra3, Sstr1, Bdkrb1, Cnr1, Adra2b, Hrh1, Lep, Gcgr, Ptger2, Cnr2, Tbxa2r, Ltb4r1, Glrb, Tshr, Cga, Grin2a, Gabrb1, P2ry13, Adrb3, Gabrp, Adra1b, Ghsr, Avpr1b, Chrna4, S1pr4, Ednra, Hrh2, P2ry10, Prlr, Grin2b, Gipr, Gabrq, P2rx5, Gabrb3, Cckbr, Gabbr2, Adora2b, Adra1d, P2ry6, Pth1r, Tacr1, Taar1, Grm3, Mc5r, Crhr1, Chrna9, Mchr1, Fpr2, Chrnb4, Htr4, S1pr3, Adra2a, P2ry2, Grik4, Grin2d, Grin3a, Lpar2, Gzma, Ltb4r2, Cysltr2, Grik3, Grin2c, Adora2a, Grin1, Ptafr, C5ar1 |
|  |  | hypertrophic_cardiomyopathy_hcm | -1.43668 | 0.024 | Il6, Ryr2, Itgb7, Itga2b, Itga4, Myl2, Myh6, Cacnb3, Itga2, Itga10, Cacna2d1, Itga7, Cacng2, Cacna1f, Tpm2, Itgb3, Tgfb2, Cacna2d2, Itga11 |
|  |  | adipocytokine_signaling_pathway | -1.43371 | 0.018 | Prkcq, Adipoq, Pck1, Nfkbie, Lep, Rxrg, Mapk10, G6pc2, Tnfrsf1b, G6pc |
|  |  | aldosterone_regulated_sodium_reabsorption | -1.42333 | 0.037 | Scnn1b, Fxyd4, Pik3cd, Atp1a3, Ins2, Prkcb, Pik3cg |
|  |  | dilated_cardiomyopathy | -1.4182 | 0.018 | Ryr2, Pln, Itgb7, Itga2b, Itga4, Myl2, Myh6, Cacnb3, Itga2, Itga10, Cacna2d1, Itga7, Cacng2, Cacna1f, Adcy5, Tpm2, Adcy7, Itgb3, Tgfb2, Cacna2d2, Itga11 |
|  |  | mapk_signaling_pathway | -1.39649 | 0.002 | Cacna1b, Cacna1e, Pla2g2d, Il1a, Fgf18, Pla2g4a, Cacna1g, Rasgrp1, Mapk8ip2, Mapk11, Mapk10, Rac2, Pla2g2f, Map4k1, Cacnb3, Ptprr, Cacna1h, Fasl, Fgf1, Fgf11, Ptpn7, Rasgrp4, Prkcb, Rac3, Fgf2, Fgf9, Hspa1a, Dusp14, Cacna2d1, Nr4a1, Hspa1l, Gadd45g, Fgf16, Cacng2, Cacna1f, Fos, Cacna1i, Tgfb2, Cacna2d2, Dusp1, Fgf22, Rasgrf1, Map3k8, Fgf7, Gadd45a, Nfkb2, Mras, Gadd45b, Flnc, Rasgrp2, Il1b, Il1r1, Nfatc4 |
|  |  | long_term_depression | -1.39378 | 0.039 | Pla2g2d, Nos1, Pla2g4a, Ryr1, Gnao1, Pla2g2f, Prkcb, Gucy1a2, Crhr1 |
|  |  | complement_and_coagulation_cascades | -1.39302 | 0.039 | Cfd, Cfi, Bdkrb1, C8b, Masp2, C6, C4b, C2, Cd55, C3, Fga, Serpina1e, F5, Serpine1, Thbd, C5ar1, Bdkrb2, C1s1, Cd59b, Serping1, C1qb, F8, C7, Cd46, C1qc, F10, C3ar1, F12, F9, Fgg, Plat |
|  |  | fc_gamma_r_mediated_phagocytosis | -1.3797 | 0.025 | Syk, Lat, Wasf1, Pla2g4a, Pla2g4f, Dock2, Rac2, Ptprc, Fcgr4, Pik3cd, Prkcb, Was, Pik3cg, Amph, Hck, Fcgr1, Inpp5d, Marcksl1, Plcg2, Ncf1, Wasf3, Pak1, Pla2g4b, Dnm3, Fcgr2b, Lyn, Pip4k2b, Vav1, Asap1, Pla2g6, Rps6kb2 |
|  |  | jak_stat_signaling_pathway | -1.32393 | 0.029 | Il6, Il7r, Osm, Il9r, Il15, Lep, Mpl, Ifng, Tslp, Il21r, Csf3r, Csf3, Il5ra, Il2rb, Pik3cd, Prlr, Pik3cg, Il2ra, Il2rg, Il23a, Il12rb2, Il10ra, Ctf1, Socs1, Il4, Stat1, Lif, Jak2, Ifnar2, Csf2ra, Socs3, Il4ra |
|  |  | drug_metabolism_cytochrome_p450 | 1.350455 | 0.049 | Adh4, Aldh3b3, Ugt2b1, Cyp2b10, Ugt2b35, Cyp1a2, Adh7, Ugt2a3 |
|  |  | fatty_acid_metabolism | 1.398043 | 0.049 | Adh4, Adh7, Aldh1b1, Acat3, Ehhadh, Eci2, Acat1, Acsl4, Cpt1c, Gcdh, Aldh9a1, Acadvl, Acadsb |
|  |  | ribosome | 1.499421 | 0.010 | Rpl3l, Rpl24, Rpl17, Rpl22l1, Rpl30, Rps10, Rpl7a, Rpl39, Rps7, Rpl31, Rpl15, Rpl27a, Rsl24d1, Rpl26, Rpl18a, Rpl27, Rps12, Rpl4, Rps15a, Rpl11, Rpl7, Rplp0, Rpl6, Rpl34, Rps6, Rpsa, Rpl28, Rps3, Rpl9, Rps27l, Rpl5, Rps26, Rps8, Rps5, Rpl23a, Rpl10a, Rps17, Rps4x, Fau, Rplp1, Rps18, Rps24, Rpl10, Rps27a, Rpl3, Rps16, Rps21, Rpl13, Rps25, Rps2, Rpl37a, Rpl12 |
| ^a^GSEA analysis was conducted by R software using package "fgsea" and "msigdbr". | | | | | |
| ^b^NES, normalized enrichment score | | |  |  |  |
